# Effective cellular and neutralizing immunity against SARS-CoV-2 after mRNA booster vaccination is associated with pDC and B cell activation

**DOI:** 10.3389/fimmu.2025.1580448

**Published:** 2025-05-12

**Authors:** Dorit Fabricius, Carolin Ludwig, Matthias Proffen, Janina Hägele, Judith Scholz, Christiane Vieweg, Immanuel Rode, Simone Hoffmann, Sixten Körper, Hubert Schrezenmeier, Bernd Jahrsdörfer

**Affiliations:** ^1^ Department of Pediatrics and Adolescent Medicine, Ulm University Medical Center, Ulm, Germany; ^2^ Department of Transfusion Medicine, Ulm University, Ulm, Germany; ^3^ Institute for Clinical Transfusion Medicine and Immunogenetics, German Red Cross Blood Transfusion Service Baden-Württemberg – Hessen and University Hospital Ulm, Ulm, Germany

**Keywords:** SARS-CoV-2, COVID-19, mRNA vaccine, vector vaccine, Comirnaty, Spikevax, Vaxzevria, Omicron

## Abstract

**Introduction:**

The emergence of SARS-CoV-2 variants of concern (VOCs), particularly Omicron, has challenged the efficacy of initial COVID-19 vaccination strategies. Booster immunizations, especially with mRNA vaccines, were introduced to enhance and prolong immune protection. However, the underlying mechanisms of humoral and cellular immunity induced by homologous versus heterologous vaccination regimens remain incompletely understood. This study aimed to elucidate the immune responses, including B cell, plasmacytoid dendritic cell (pDC), and T cell activation, following mRNA booster vaccination.

**Methods:**

In a longitudinal cohort study, 136 individuals received three different vaccination regimens: homologous mRNA, heterologous vector-mRNA-mRNA, or heterologous vector-vector-mRNA vaccinations. Serum and peripheral blood mononuclear cells (PBMCs) were collected at multiple time points up to 64 weeks after initial vaccination. Anti-SARS-CoV-2 IgG titers and neutralization capacity against the wildtype virus and Omicron variant were measured using ELISA and cPass assays. Cellular immunity was assessed by IFN-γ release assays, and flow cytometry was employed to analyze B cell and pDC frequencies, viability, and activation markers. Functional pDC-mediated T cell activation was evaluated in mixed lymphocyte cultures.

**Results:**

mRNA booster vaccination stabilized high anti-SARS-CoV-2 IgG titers and neutralizing activity against wildtype virus across all regimens, with the homologous mRNA group showing the highest antibody titers and Omicron neutralization capacity. Peripheral B cell frequencies and activation markers (MHC class I/II, CD86) were significantly upregulated post-booster. pDCs demonstrated enhanced antigen-presenting capacity and significantly promoted SARS-CoV-2-specific T cell IFN-γ responses *in vitro*. Despite differences in humoral responses between regimens, breakthrough infection rates up to 25 weeks post-booster were comparable across cohorts, suggesting compensatory mechanisms via cellular immunity.

**Discussion:**

Our findings highlight the pivotal role of pDCs and T cells in sustaining effective immunity following mRNA booster vaccination. While homologous mRNA regimens induce superior humoral responses, robust cellular immunity in heterologous regimens may balance protection levels against breakthrough infections. The study underscores the importance of integrated humoral and cellular immune responses, suggesting potential for optimized booster strategies and pDC-targeted vaccine designs to enhance long-term protection against SARS-CoV-2 and emerging variants.

## Introduction

The COVID-19 pandemic, caused by SARS-CoV-2, has become one of the most significant global health crises in recent history. First identified in late 2019, SARS-CoV-2 has caused millions of infections and deaths, disrupted economies, and strained healthcare systems worldwide. Within three years, the virus evolved five major variants of concern (VOCs) (Alpha, Beta, Gamma, Delta, and Omicron), characterized by enhanced infectivity, transmissibility and immune evasion mechanisms (1–3). The most widely spread variant Omicron (B.1.1.529) acquired more than 50 characteristic mutations in different motifs of the spike protein (4), before on May 5–2023 the World Health Organization declared COVID-19 to be no longer a pandemic-level threat (https://www.who.int/news/item/05-05-2023-statement-on-the-fifteenth-meeting-of-the-international-health-regulations-(2005)-emergency-committee-regarding-the-coronavirus-disease-(covid-19)-pandemic).

Efforts to mitigate the pandemic’s impact relied heavily on the rapid development and deployment of vaccines. Among these, mRNA vaccines such as BNT162b2 (Pfizer-BioNTech) and mRNA-1273 (Moderna) represented a breakthrough in vaccinology. Unlike traditional vaccines, mRNA vaccines deliver genetic instructions to host cells to produce the viral spike protein, eliciting a robust immune response (5). These vaccines demonstrated high efficacy in clinical trials, reducing symptomatic infections, hospitalizations, and deaths during the early phases of the pandemic (6–10). However, the emergence of SARS-CoV-2 variants introduced challenges due to mutations in the spike protein. Such mutations allowed VOCs to partially evade antibody-mediated immunity, reducing the effectiveness of initial vaccine doses (11, 12). Booster vaccinations with updated mRNA sequences were introduced as a critical strategy to restore and sustain immunity, ensuring continued protection against severe disease and hospitalization with mutated virus strains (13). Understanding the immunological mechanisms activated by these boosters is essential to optimizing vaccination schedules and formulations for long-term protection.

Initially, two approaches have been employed in the context of COVID-19 vaccination strategies: homologous and heterologous vaccination. Homologous vaccination involves administering the same vaccine type for both the initial (prime) and subsequent (boost) doses. In contrast, heterologous vaccination - also known as a “mix-and-match” approach - utilizes different vaccine types for the prime and boost doses (14–16). Emerging evidence suggests that heterologous vaccination regimens can elicit robust immune responses. Early studies during the COVID-19 pandemia indicated that a heterologous prime-boost schedule combining an adenoviral vector vaccine (such as ChAdOx1 nCoV-19) with an mRNA vaccine (like BNT162b2) induced higher levels of neutralizing antibodies and stronger T-cell responses compared to homologous regimens (10, 17–19). Additionally, research showed that heterologous booster vaccinations enhanced neutralizing responses against variants of concern, including the Omicron variant (10, 20). These findings suggested different vaccination strategies may have significant impact on vaccine-induced immunity, particularly in the context of emerging SARS-CoV-2 variants.

Humoral immunity, mediated by B cells, plays a pivotal role in the immune response to SARS-CoV-2. Upon antigen exposure, naive B cells differentiate into plasma cells, which secrete antibodies, or into memory B cells, which provide rapid and robust responses upon re-exposure (21, 22). Antibodies, particularly IgG, target the viral spike protein, blocking its interaction with the ACE2 receptor and preventing viral entry into host cells (23). Early studies demonstrated that mRNA vaccines induced high titers of neutralizing antibodies, correlating with protection against symptomatic disease (24, 25). However, antibody levels naturally declined over time, a phenomenon that called for booster doses to maintain protective titers (26). In addition to quantity, the quality of anti-viral antibody responses is crucial for their effectivity. Memory B cells undergo affinity maturation in germinal centers, resulting in the production of antibodies with increased specificity and binding strength (27, 28). This process is particularly important in the context of SARS-CoV-2 variants, where higher-affinity antibodies are more likely to neutralize mutated spike proteins. Booster vaccinations were shown to enhance both the quantity and quality of the humoral response, particularly in individuals receiving mRNA vaccines (10, 29–31).

While antibodies are essential for neutralizing extracellular viruses, cellular immunity provides an additional layer of protection by targeting infected cells and preventing viral replication (32). T cells, particularly CD4^+^ helper and CD8^+^ cytotoxic T cell subsets, are activated during vaccination (29, 30, 33). CD4^+^ T cells facilitate B cell activation and antibody production, while CD8^+^ T cells are able to directly kill virus-infected cells. Notably, T cell responses are less affected by mutations in the spike protein, as many T cell epitopes appear to be conserved across SARS-CoV-2 variants (29, 30). This resilience makes cellular immunity a critical component of long-term protection. mRNA vaccines have demonstrated their ability to induce robust T cell responses, which are characterized by the production of cytokines such as interferon-gamma (IFN-γ). These cytokines enhance antiviral activity of T cells and natural killer cells, further amplifying the antiviral immune response (29, 30). In addition to providing protection against severe disease, cellular immunity plays a compensatory role in individuals with waning antibody levels or partial immune escape by VOCs (34, 35). The durability of T cell responses post-vaccination has therefore been a key focus of prior and ongoing vaccine research.

Plasmacytoid dendritic cells (pDCs) are a unique subset of immune cells known for their ability to produce large quantities of type I interferons such as IFN-α during viral infections (36, 37). These cytokines activate antiviral pathways and enhance the adaptive immune response by promoting T cell activation and B cell differentiation (38). pDCs also serve as antigen-presenting cells, linking the innate and adaptive arms of the immune system (39, 40). Therefore, in the context of SARS-CoV-2 infection and vaccination, pDCs are also hypothesized to play a crucial role in shaping the immune response, particularly by enhancing T cell-mediated immunity and facilitating memory B cell maturation (36).

The present study aims at elucidating the immunological mechanisms underpinning effective cellular and humoral immunity against SARS-CoV-2 following mRNA booster vaccination over a longer period of time. By analyzing serum and PBMC samples from diverse vaccination cohorts, our research investigates the stability of SARS-CoV-2-neutralizing IgG titers, the activation level and viability of B cells, the durability of T cell responses and the role of pDCs for the latter. Moreover, it evaluates differential effects of homologous and heterologous vaccination regimes on immune protection against SARS-CoV-2 in general and its most wide-spread variant Omicron, while directly comparing their impact on breakthrough infections.

## Materials and methods

### Vaccination cohorts

For the present cohort study, we used serum and PBMCs from up to 103 female and 33 male individuals (**Supplementary Table 1**). Due to the design of cohort studies both gender and age imbalances in the cohorts could not be entirely prevented. Regarding gender, we were not able to identify significant differences between males and females when comparing the analyte with the highest number of data points available, which was SARS-CoV-2 IgG (**Supplementary Figure 1**). With regard to the minor age differences in the cohorts of the present study, we refer to our recent findings in nursing home residents, which demonstrated that robust neutralizing immune responses following full vaccination with the mRNA vaccine BNT162b2 were achieved in a large cohort of individuals spanning a broad age range from 18 to 98 years (41, 42). From all individuals included into the present study up to 68 received a homologous mRNA vaccination regime (m^3^), either with Comirnaty (BioNTech/Pfizer, Mainz, Germany), or with Spikevax (Moderna, Cambridge, Massachusetts, USA). Up to 55 individuals received a heterologous vaccination regime involving one vaccination with the vector vaccine Vaxzevria (AstraZeneca, Cambridge, UK) and two vaccinations with an mRNA vaccine (Comirnaty or Spikevax, vm^2^). Up to 13 individuals received a heterologous vaccination regime involving two vaccinations with the vector vaccine Vaxzevria and one mRNA booster vaccination (v^2^m). The interval between first and second vaccination was 3–6 weeks if only mRNA vaccines were used, 5–14 weeks for primary vaccination with a vector vaccine and secondary vaccination with an mRNA vaccine, and 4–13 weeks for primary and secondary vaccination with a vector vaccine. Median time point for booster (3^rd^) vaccination in our study was 37.1 weeks after first vaccination (range 29.4-46.1 weeks) in the homologous mRNA cohort (m^3^), 34.0 weeks after first vaccination (range 24.4-44.6 weeks) in the heterologous cohort with two mRNA vaccinations (vm^2^), and 34.0 weeks after first vaccination (range 21.0-38.3 weeks) in the heterologous cohort with one mRNA vaccination (v^2^m). Median age of all individuals was 49 years (range 18–78 years). Only individuals with no medical history or medications affecting their systemic immunity were included. The use of blood from subjects before and after vaccination was approved by the Institutional Review Board of Ulm University.

### Serum and PBMC isolation for direct analyses and cryopreservation

After having obtained informed consent, 6 ml blood was collected from each donor in serum collection tubes (Vacuette, Greiner Bio-One GmbH, Frickenhausen, Germany) for serological testing and neutralization tests. Sera were aliquoted after centrifugation and cryopreserved at -20°C until further use or transferred to -80 °C for long-term storage. PBMCs for flow cytometric measurements were obtained from 10–20 ml heparin blood (BD glass Vacutainer with sodium heparin, Becton Dickinson, New Jersey, USA) at the respective time points by BioColl density gradient centrifugation.

### Quantitative measurement of anti-SARS-CoV-2 IgG

The quantitative anti-SARS-CoV-2 IgG ELISA (QuantiVac, EUROIMMUN, Lübeck, Germany) is based on a classical sandwich ELISA, which detects IgG directed against the S1 domain of the SARS-CoV-2 spike (S) protein. The quantitative values were calculated using six calibrators and various pre-dilutions, which can be selected using the fully automated EUROIMMUN Analyzer I-2P (EUROIMMUN, Lübeck, Germany). Where necessary, conversion from RU/ml to BAU/ml was achieved by multiplication with the factor 3.2. Values ≥ 32.0 BAU/ml were considered borderline, values ≥ 35.2 BAU/ml were considered positive.

### cPass neutralization assay

This surrogate SARS-CoV-2 neutralization test (GenScript, Nanjing, China) is a blocking ELISA, which indirectly detects virus-neutralizing antibodies. If such antibodies are present in the serum, they interfere with the binding of horseradish peroxidase (HRP)-conjugated SARS-CoV-2 receptor binding domain (RBD) fragments to its receptor angiotensin-converting enzyme 2 (ACE2), which is immobilized on an ELISA microtiter plate surface. Two different types of RBD fragments were used: The original wild type SARS-CoV-2-spike RBD, and the RBD representing the variant of concern Omicron (B.1.1.529), which carries the mutations G339D, S371L, S373P, S375F, K417N, N440K, G446S, S477N, T478K, E484A, Q493R, G496S, Q498R, N501Y and Y505H. In a pre-incubation step, RBD fragments, conjugated to horseradish peroxidase (HRP-RBD), get neutralized by samples and controls containing neutralizing antibodies, and only unbound HRP-RBD might be captured by ACE2 on the microplate in the second step. The reaction of the substrate TMB by HRP results in a color development whose intensity inversely correlates with the amount of neutralizing antibodies. Neutralization capacities >30% were considered as positive, neutralization capacities >70% were considered as strong.

### Interferon-gamma (IFN-γ) release assay (IGRA)

The SARS-CoV-2 Interferon-gamma (IFN-γ) release assay (IGRA, EUROIMMUN, Lübeck, Germany) detects the T cell-mediated immune response to the SARS-CoV-2 spike antigen in heparin blood. To that purpose, 500µl heparinized whole blood from various individuals was incubated for 20–24 hours at 37°C, 5% CO_2_ and 95% humidity in Quan-T-Cell “T”-tubes (EUROIMMUN, Lübeck, Germany). “T”-tubes are coated with peptide antigens from the SARS-CoV-2 S1 domain, so that incubated cells are exposed to the antigens. IFN-γ released by SARS-CoV-2-specific T cells can subsequently be detected in the supernatants using the SARS-CoV-2 Quan-T-Cell ELISA (EUROIMMUN, Lübeck, Germany) on the EUROIMMUN Analyzer I-2P according to the manufacturer`s instructions. Results are given in mIU/ml.

### Flow cytometric analysis of cryopreserved PBMCs

Cryopreserved PBMCs from a total of 56 individuals and collected at four different time points (before 1^st^ vaccination as well as 0, 1 and 3 months after booster vaccination) were thawed and incubated overnight at 0.5 x 10^6^ cells per well in a 48-well plate in 333µl AIM-V medium (Gibco Life Technologies, Paisley, UK) supplemented with 10 ng/ml IL-3 and 100ng/ml IL-21 (Miltenyi Biotec, Bergisch Gladbach, Germany) at 37°C. After 20 hours, 5 μg/ml Brefeldin A (BioLegend, San Diego, California, USA) was added and after another 4 hours the cells were fixed and stained either with or without permeabilization (Inside Stain Kit, Miltenyi Biotec, Bergisch Gladbach, Germany). Staining panel 1 was used on permeabilized cells and included anti-BDCA-2 FITC (Miltenyi Biotec, Bergisch Gladbach, Germany), anti-CD19 APC-Cy7, anti-IL-10 PE-Cy7, anti-CD80 BV421 (all from BioLegend, San Diego, California, USA), anti-GrB APC (Invitrogen, Carlsbad, California, USA) and anti-CD86 PE stainings (BD Pharmingen, San Diego, California, USA). Staining panel 2 was used on non-permeabilized cells and included anti-BDCA-2 PE, anti-CD154 APC-Vio770 (both from Miltenyi Biotec, Bergisch Gladbach, Germany), anti-CD19 PECy7 (BioLegend, San Diego, California, USA), anti-MHC class I FITC (BD Pharmingen San Diego, California, USA) and anti-MHC class II PerCP (BD Biosciences, San Jose, California, USA). Cells were analyzed on a BD FACSCelesta (BD, San Jose, California, USA), data analysis was carried out using FlowJo software version 10.5.3 (BD, Ashland, Oregon, USA.

### Mixed lymphocyte cultures with pDCs

For pDC isolation, PBMCs from 12 healthy donors were used who had received three doses of the above mentioned COVID-19 vaccines in various combinations between December 2020 and October 2022. Three of the pDC donors had no NAT-confirmed infection with SARS-CoV-2, eight donors had one infection and one donor had two infections. On day 0, 120-140ml blood from each donor was collected in heparin-coated glass tubes (BD Vacutainer, Becton Dickinson, New Jersey, USA). PBMCs were separated by density-gradient centrifugation using Lymphosep lymphocyte separation solution (Biowest, Missouri, USA), followed by incubation with 200µl CliniMACS CD304 (BDCA-4) reagent (Miltenyi Biotec, Bergisch Gladbach, Germany). Then, pDCs were isolated by positive magnetic selection using the Quadro MACS multistand and MACS LS columns (Miltenyi Biotec, Bergisch Gladbach, Germany) according to the manufacturer`s instructions as previously described (43). Isolated pDCs were counted and incubated in serum-free AIM-V medium (Gibco Life Technologies, Paisley, UK), supplemented with 10 ng/ml IL-3 (Miltenyi Biotec, Bergisch Gladbach, Germany). 200 x 10^3^ pDCs were incubated for three days with or without 0.8 µg/ml COVID-19 peptide vaccine (Novavax, Gaithersburg, Maryland, USA) in a total volume of 200 µl/well on 96-well V-bottom plates (Greiner Bio-one, Frickenhausen, Germany). Non-pDCs (negative fraction) served as control and were incubated at the same cell concentration as pDCs for three days with 0.8 µg/ml COVID-19 peptide vaccine. On day 2, 2.5µg/ml ODN 2006 (Miltenyi Biotec, Bergisch Gladbach Germany) was added to each well to induce pDC maturation. 96-well V-bottom plates were incubated at 37°C, 5% CO_2_ and 95% humidity. The purity of the isolated pDCs was determined by staining with the lineage cocktail Lin-1 FITC (BD Biosciences, San Jose, California, USA), anti-human CD303 (BDCA-2) PE (Miltenyi Biotec, Bergisch Gladbach, Germany) and anti-human CD123 PE-Cy5 (BD Pharmingen, San Diego, California, USA) as previously described (43). Samples were analyzed on a BD FACSCelesta (BD, San Jose, California, USA), data analysis was carried out using FlowJo Software version 10.5.3 (BD, Ashland, Oregon, USA). On day 3, another 25ml heparin blood was collected from all corresponding donors and PBMCs were isolated by density-gradient centrifugation. Freshly isolated PBMCs as well as the previously incubated pDCs and non-pDCs were counted after a washing step with AIM-V medium. Then, 500 x 10^3^ freshly isolated PBMCs were incubated with 12.5 x 10^3^ pre-incubated pDCs or non-pDCs (ratio 40:1) in a total volume of 500µl AIM-V medium in Quan-T-Cell “T”-tubes (EUROIMMUN, Lübeck, Germany) to induce SARS-CoV-2-specific IFN-γ release. 500 x 10^3^ PBMCs incubated in cell-free medium containing 0.8 µg/ml Novavax served as an additional control. “T”-tubes were incubated for 20–24 hours at 37°C, 5% CO_2_ and 95% humidity. Finally, IFN-γ in the supernatant of the “T”-tubes was quantified using the SARS-CoV-2 Quan-T-Cell ELISA (EUROIMMUN, Lübeck, Germany) on the EUROIMMUN Analyzer I-2P according to the manufacturer`s instructions. Results are given in mIU/ml.

### Statistical analysis

Statistical analysis was performed using Microsoft Excel for Mac version 16.16.8 and GraphPad Prism version 9.0.0. Some of the summarized data are shown in line graphs using mean values ± SEM. In addition, summarized data in bar graphs are expressed as box plots, the medians being represented by the central horizontal lines and the interquartile ranges, the minima and the maxima being represented by the box edges and whiskers. For multiple comparisons of more than two independent data sets or more than two variables, Dunnetts’s multiple comparisons test was performed. Furthermore, Spearman correlation was used to assess the relationship between anti-spike titers and neutralization capacity.

## Results

### Booster vaccination with an mRNA vaccine results in long-term stabilization of anti-SARS-CoV-2 IgG titers with neutralizing capacities on a high level

**Figure 1:**
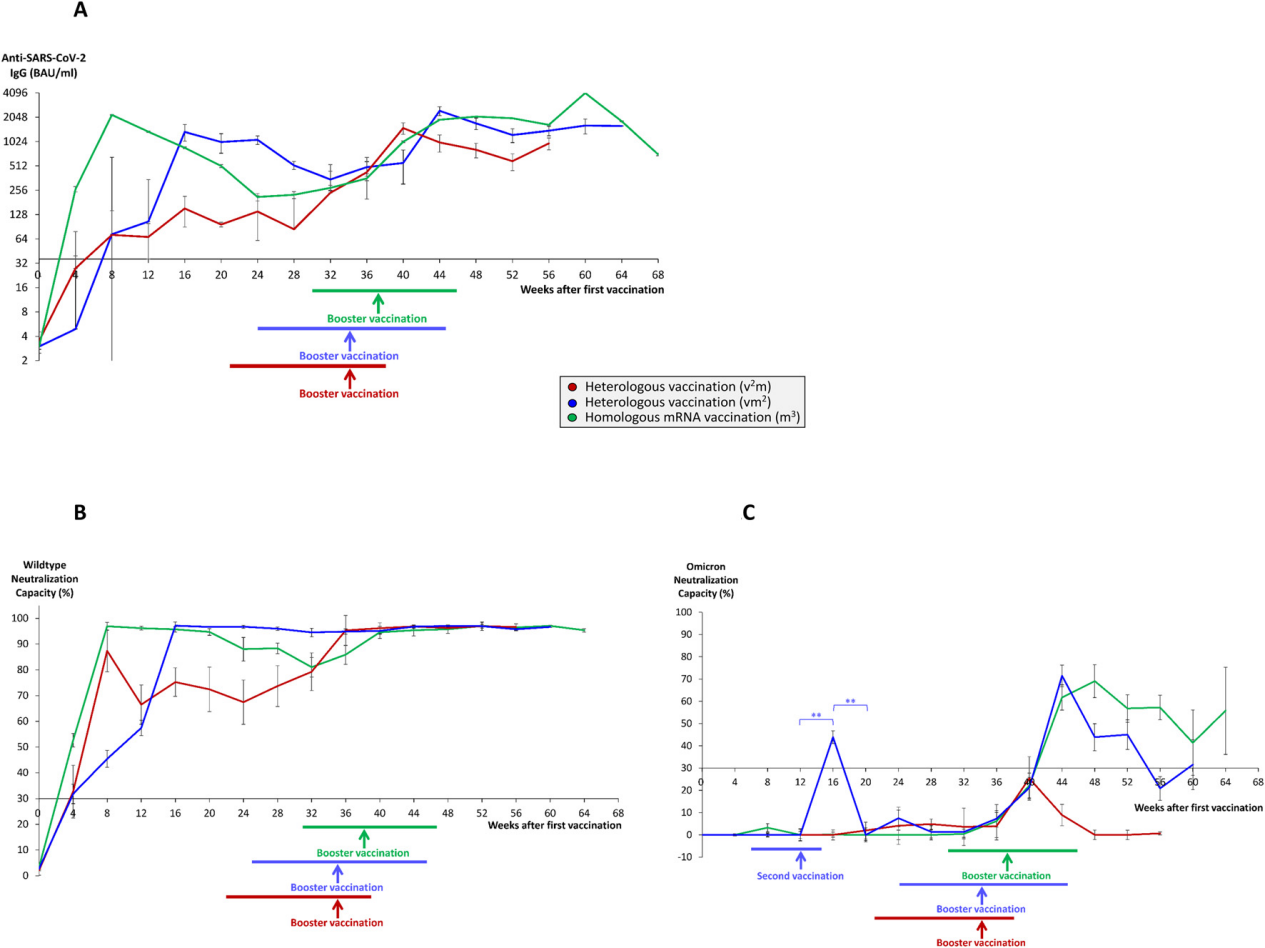
Time course of antibody and neutralization titers in homologous and heterologous anti-SARS-CoV-2 vaccination regimes. Serum samples from 68 individuals having received a homologous vaccination regime consisting of three mRNA vaccines (m^3^, green lines), 55 individuals having received a heterologous vaccination regime consisting of one vector and two mRNA vaccines (vm^2^, blue lines), and 13 individuals having received a heterologous vaccination regime consisting of two vector and one mRNA vaccine (v^2^m, red lines), were collected at different time points after 1^st^ vaccination as indicated. 2^nd^ vaccination was generally given 3–4 weeks after 1^st^ vaccination in mRNA^3^ vaccinees (median 4 weeks) and 6–14 weeks (median 12 weeks) after 1^st^ vaccination in vm^2^ and v^2^m regimes. Booster vaccination refers to the 3^rd^ vaccination with an mRNA vaccine. Colored horizontal lines below the x-axis indicate the time range of booster vaccination after first vaccination, colored vertical arrows indicate median time points of booster vaccination after first vaccination. Line graphs show average results for **(A)** anti-SARS-CoV-2 IgG titers, **(B)** wildtype SARS-CoV-2 neutralization capacities, and **(C)** Omicron neutralization capacities. Error bars indicate SEM, the significance level was **p < 0.005. Anti-SARS-CoV-2 IgG titers >35.2 BAU/ml and neutralization capacities >30% were considered as positive, neutralization capacities >70% were considered as strong.

A total of 136 individual vaccinees received 3 different vaccination regimes. The first regime consisted of 3 shots of one of the two mRNA vaccines BNT162b2 from BioNTech/Pfizer (Comirnaty) or mRNA-1273 from Moderna (Spikevax) (m^3^ regime, green color). The second regime consisted of one shot of the vector vaccine ChAdOx1-nCoV-19 from AstraZeneca (Vaxzevria), followed by two shots of an mRNA vaccine (vm^2^ regime, blue color), and the third regime of two shots of the vector vaccine, followed by one booster shot of an mRNA vaccine (v^2^m regime, red color). Vaccinees were screened for their humoral anti-SARS-CoV-2 immune response over a time period of up to 64 weeks after 1^st^ vaccination (**Figure 1**). Since our first study from 2021 found BNT162b2 and mRNA-1273 to be equally effective in inducing a rapid anti-SARS-CoV-2 humoral response (10), we did not differentiate between both vaccines for our current analysis. End points analyzed included anti-SARS-CoV-2 IgG titers on the one hand, and neutralization capacities against the wildtype virus and the Omicron variant as the latest clinically relevant VOC in this pandemic on the other hand.

**Figure 2:**
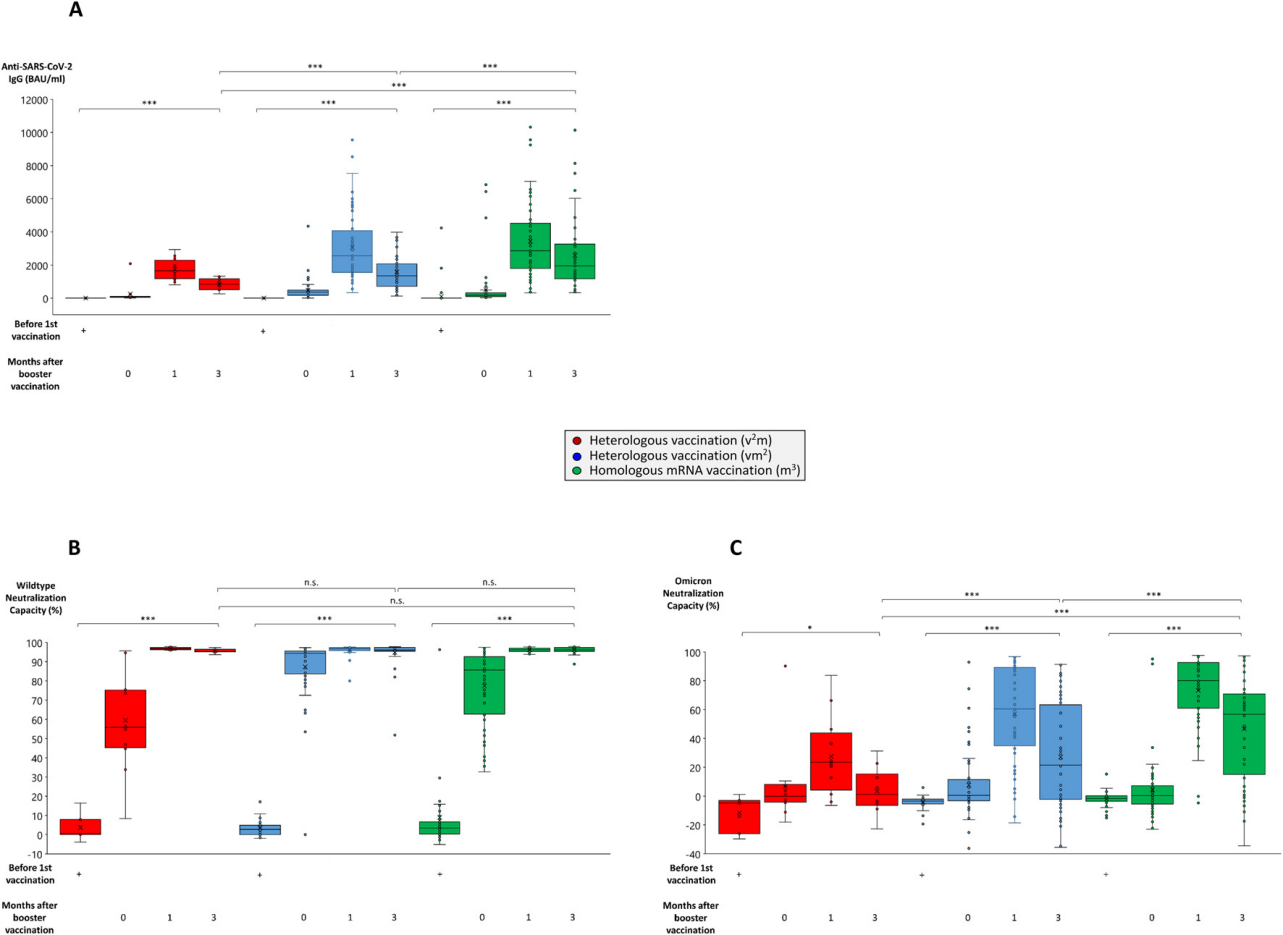
Comparison of antibody and neutralization titers in homologous and heterologous anti-SARS-CoV-2 vaccination regimes. Serum samples from up to 64 individuals having received a regime with three mRNA vaccines (m^3^, green bars), up to 53 individuals having received a regime with one vector and two mRNA vaccines (vm^2^, blue bars), and up to 13 individuals having received a regime with two vector and one mRNA vaccine (v^2^m, red bars), were collected before 1^st^ vaccination and 0, 1 and 3 months after mRNA booster vaccination (3^rd^ vaccination) as indicated. Box blots show **(A)** anti-SARS-CoV-2 IgG titers, **(B)** wildtype SARS-CoV-2 neutralization capacities, and **(C)** Omicron neutralization capacities. Box central horizontal lines indicate medians, box borders represent IQR, whiskers indicate minima and maxima. Significance levels were ***p < 0.0005 and *p < 0.05. IQR, interquartile range; n.s., not significant.

Most strikingly, within 4 to 10 weeks after booster vaccination, anti-SARS-CoV-2 IgG titers reached all-time highs in the respective groups (in average 4079 BAU/ml in the m^3^ group, 2454 BAU/ml in the vm^2^ group and 1502 BAU/ml in the v^2^m group) (**Figure 1A**), with stable wildtype neutralization capacities close to 100% regardless of the vaccination regime (**Figure 1B**). In the further course, both anti-SARS-CoV-2 IgG titers and wildype neutralization capacities remained stable for up to 64 weeks after first vaccination. Nevertheless, three months after booster vaccination anti-SARS-CoV-2 IgG titers still showed significant differences between the three vaccination regimes with the highest mean titers found in the m^3^ group (2011 BAU/ml), followed by the vm^2^ group (1389 BAU/ml) and the v^2^m group (809 BAU/ml) (**Figure 2A**). These differences in antibody titers however did not translate into significant differences in wildtype neutralization capacities, which remained on a high level close to 100% in all vaccination groups (**Figure 2B**). Intriguingly, analysis of the Omicron neutralization capacity (including retrospective analysis of samples acquired before the first cases of Omicron were even described) demonstrated a weak, but significant peak between weeks 12 and 20 in those individuals having received the vector vaccine first, followed by an mRNA vaccine as second vaccination (**Figure 1C**, blue line). In the other both vaccine groups (v^2^m and m^3^), no comparable early signal was detected for Omicron neutralization capacity. After booster vaccination however, the vaccination groups with at least two mRNA shots (vm^2^ and m^3^) build up significant neutralization capacities against Omicron, which were stable for at least 12 weeks (**Figure 1C**, blue and green lines). In contrast, the group with two vector vaccine shots and one mRNA vaccine booster failed to reach significant protection against the Omicron variant at any time (**Figure 1C**, red line). Again, three months after booster vaccination, Omicron neutralization capacities still showed significant differences between the three vaccination regimes with the highest mean protection detected in the m^3^ group (57%), followed by the vm^2^ group (22%) and the v^2^m group (6%) (**Figure 2C**). Up to the time of booster vaccination no infections with SARS-CoV-2 had been reported in any of the vaccination groups.

### Booster vaccination with an mRNA vaccine positively impacts on peripheral B cell number and activation

**Figure 3:**
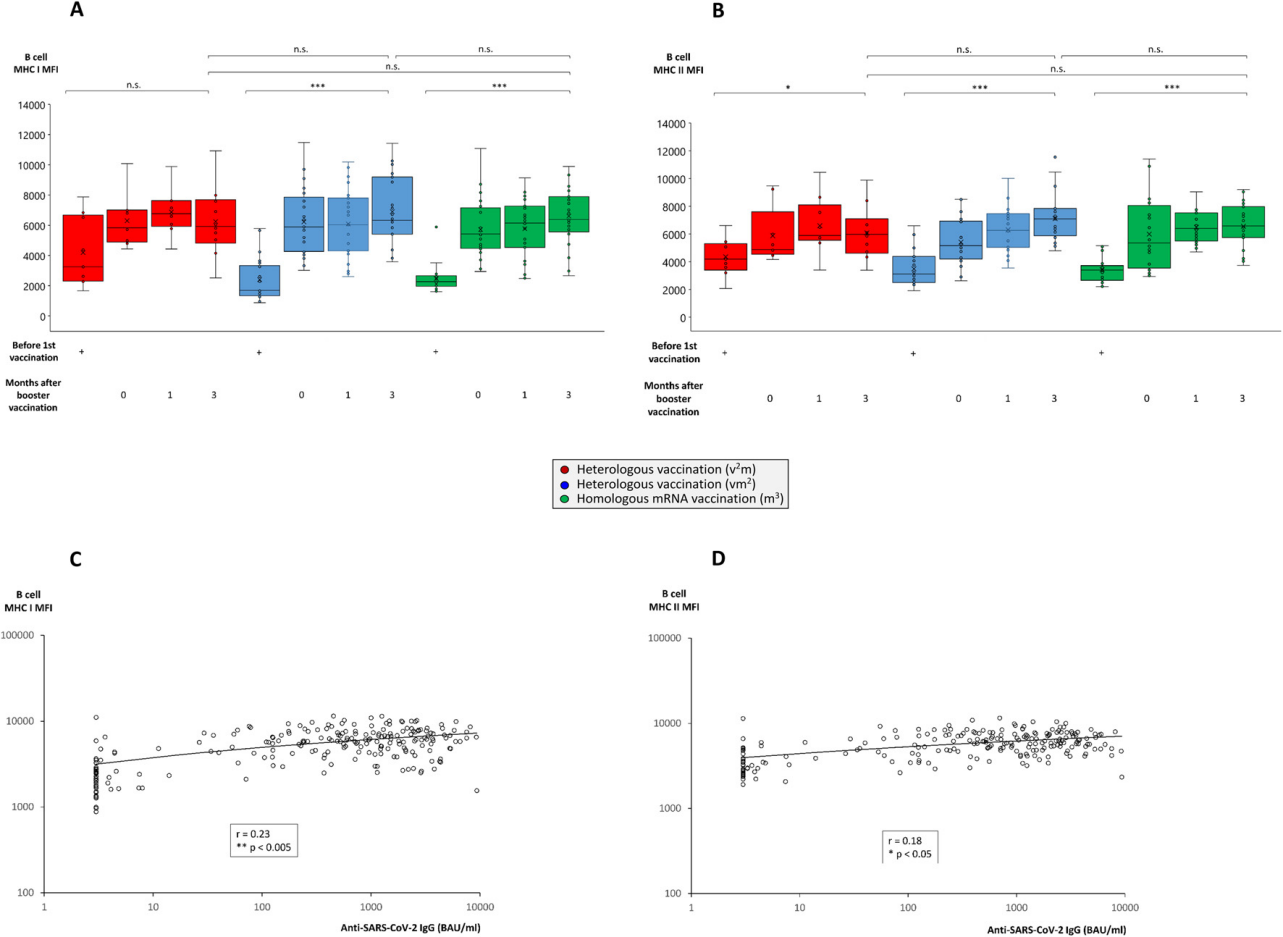
Comparison of MHC class I and II expression on B cells from individuals undergoing various anti-SARS-CoV-2 vaccination regimes. Heparin blood samples from up to 27 individuals having received a regime with three mRNA vaccines (m^3^, green bars), up to 22 individuals having received a regime with one vector and two mRNA vaccines (vm^2^, blue bars), and up to 10 individuals having received a regime with two vector and one mRNA vaccine (v^2^m, red bars), were collected before 1^st^ vaccination and 0, 1 and 3 months after mRNA booster vaccination (3^rd^ vaccination) as indicated. PBMCs were isolated and cryopreserved until further use. Then, PBMCs were thawed, B cells stained as described in the Materials & Methods section and analyzed by flow cytometry. Box blots show **(A)** MHC class I and **(B)** MHC class II expression on CD19^+^ B cells. Box central horizontal lines indicate medians, box borders represent IQR, whiskers indicate minima and maxima. Dot plots show significant correlations between anti-SARS-CoV-2 IgG titers and **(C)** MHC class I or **(D)** MHC class II expression on B cells. Significance levels were ***p < 0.0005, **p < 0.005 and *p < 0.05. IQR, interquartile range; MHC, major histocompatibility complex; n.s., not significant.

An important link between cellular and humoral immune response are antigen-specific B cells, due to their potential to differentiate into antibody-producing plasma cells upon activation and T cell help. In the current study we could demonstrate that the percentage of peripheral B cells did not significantly differ between the three vaccination groups and ranged between 4.5% and 8.0% of all PBMC. Within the vaccinations groups having received at least two mRNA vaccinations however, peripheral B cell frequency was significantly enhanced after booster vaccination compared to the time point before 1^st^ vaccination (4.5% - 5.0% before 1^st^ vaccination, 7.5% - 8.0% after booster vaccination) (**Supplementary Figure 2A**). This finding was paralleled by a slightly, but significantly higher B cell viability (87.9% - 93.6% before 1^st^ vaccination, 93.3% - 94.0% after booster vaccination) in these groups (**Supplementary Figure 2B**). More importantly, we found several parameters for B cell activation to significantly increase with ongoing vaccination progress, although no significant differences were noted between the various vaccination groups. For example, antigen-presenting molecules such as MHC class I and II (**Figures 3A, B**) and co-stimulatory molecules including CD86 and CD154 (CD40 ligand) (**Supplementary Figures 3A, B**) were strongly and significantly enhanced after booster vaccination. In line with a direct relation of B cell activation to plasma cell differentiation and antibody production, we also found a mild, but significant correlation between the expression of MHC class I and II molecules and plasma titers of anti-SARS-CoV-2 IgG (**Figures 3C, D**).

### Induction of a strong pan-T cell IFN-γ response after booster vaccination in vaccination regimes consisting of at least two mRNA shots

**Figure 4:**
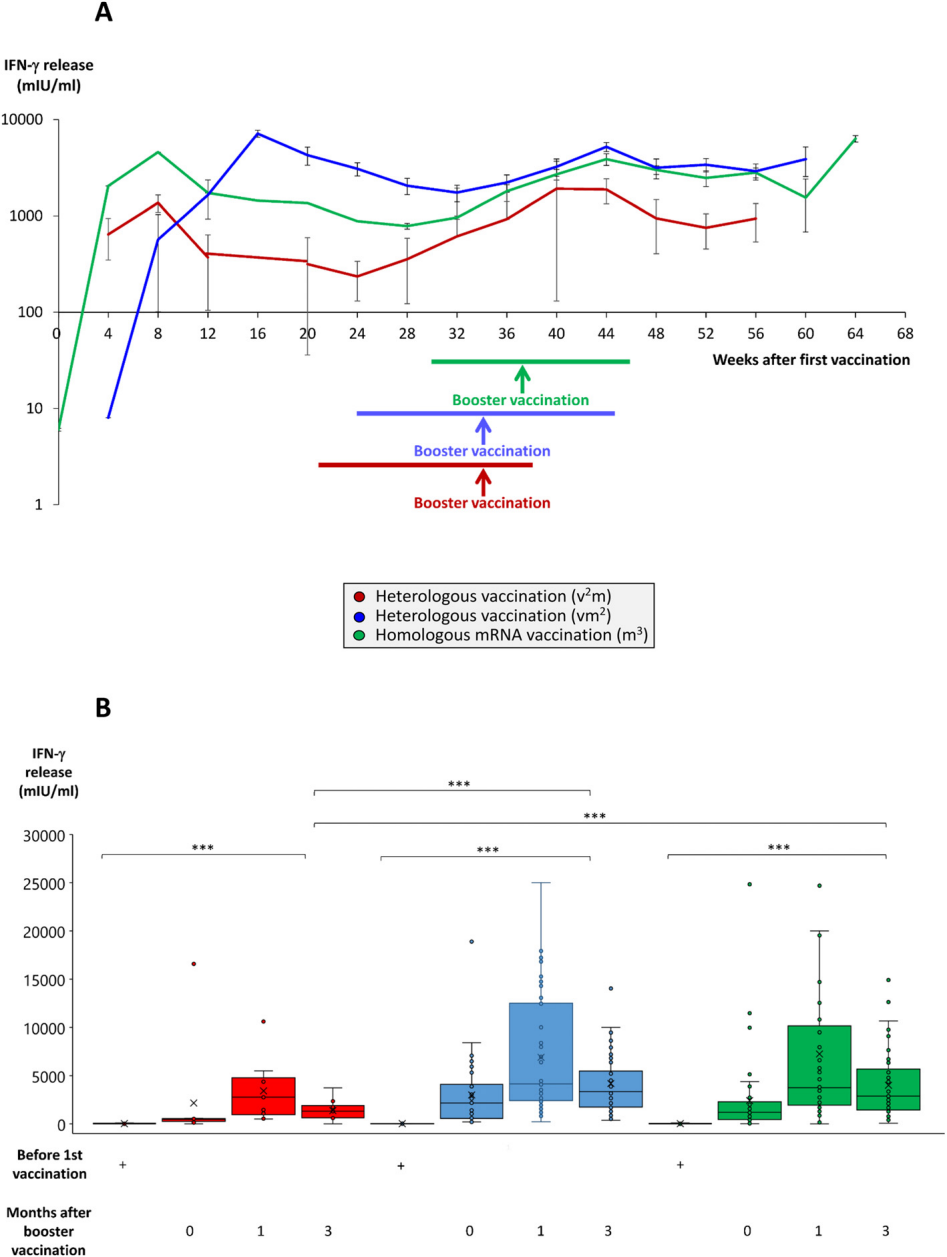
Time course and comparison of SARS-CoV-2-specific T cell response after booster vaccination in homologous and heterologous vaccination regimes. Heparinized whole blood from up to 59 individuals having received a regime with three mRNA vaccines (m^3^, green line/bars), up to 53 individuals having received a regime with one vector and two mRNA vaccines (vm^2^, blue line/bars), and up to 13 individuals having received a regime with two vector and one mRNA vaccine (v^2^m, red line/bars), was collected at different time points after 1^st^ vaccination as indicated. Heparin blood samples were incubated overnight with a SARS-CoV-2-spike peptide mixture as described in the Methods Section. Then, supernatants were harvested and IFN-γ concentrations measured by ELISA. **(A)** Line graph shows average time course of IFN-γ release in the three vaccination regimes. **(B)** Box blots show median IFN-γ release in various vaccination regimes at different time points after 1^st^ vaccination as indicated. Box central horizontal lines indicate medians, box borders represent IQR, whiskers indicate minima and maxima. Significance level was ***p < 0.0005. IFN-γ, interferon-gamma; IQR, interquartile range.

We also compared the three different vaccination cohorts with respect to a SARS-CoV-2-specific IFN-γ secretory response of pan-T cells as an important representative aspect of the cellular immune response. Before booster vaccination, IFN-γ release capacities of pan-T cells reached a nadir between weeks 24 and 32 after 1^st^ vaccination (**Figure 4A**). The mRNA booster vaccination strongly reactivated IFN-γ responses in all vaccination regimes, although the T cell response remained on a significantly lower level in the vaccination regime with two vector vaccinations followed by one mRNA booster at all times (**Figure 4A**, red line). In contrast, vaccination regimes consisting of two or three mRNA shots, provided stable and high IFN-γ release levels up to week 60 after 1^st^ vaccination (**Figure 4A**, blue and green lines). Three months after booster vaccination, IFN-γ release in the vaccination regimes with two or three mRNA shots still ranged at significantly higher levels (in average 2468 mIU/ml in the m^3^ group and 3173 mIU/ml in the vm^2^ group) than in completely unvaccinated individuals or in individuals vaccinated with two vector and one mRNA shot (in average 1297 mIU/ml in the v^2^m group) (**Figure 4B**).

### Booster vaccination with an mRNA vaccine strongly enhances plasmacytoid dendritic cell antigen-presenting and T cell-activating capacity, but may also increase their immunoregulatory potential

**Figure 5:**
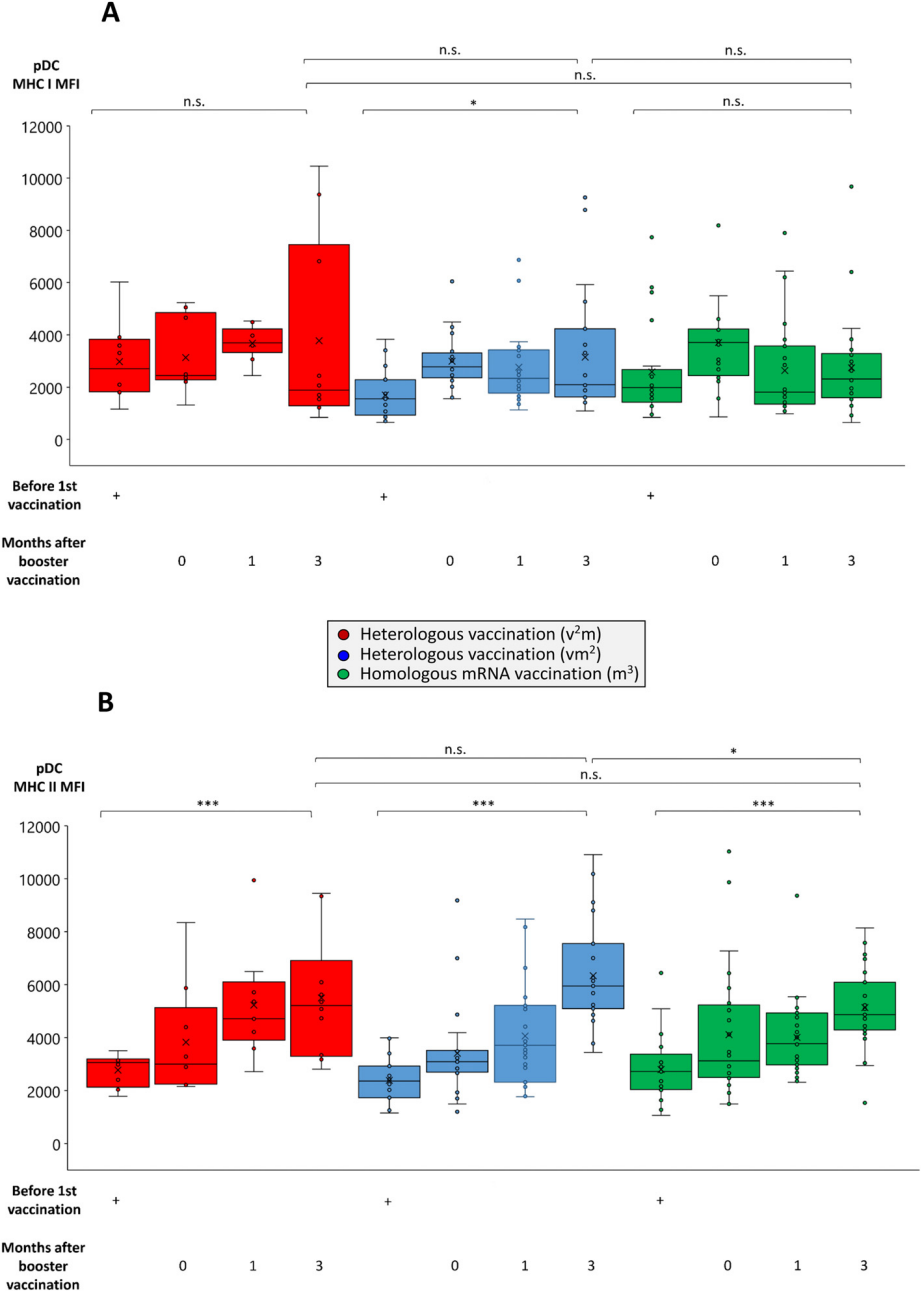
Comparison of MHC class I and II expression on pDCs from individuals undergoing various anti-SARS-CoV-2 vaccination regimes. Heparin blood samples from up to 27 individuals having received a regime with three mRNA vaccines (m^3^, green bars), up to 22 individuals having received a regime with one vector and two mRNA vaccines (vm^2^, blue bars), and up to 10 individuals having received a regime with two vector and one mRNA vaccine (v^2^m, red bars), were collected before 1^st^ vaccination and 0, 1 and 3 months after mRNA booster vaccination (3^rd^ vaccination) as indicated. PBMCs were isolated and cryopreserved until further use. Then, PBMCs were thawed, pDCs stained as described in the Materials & Methods section and analyzed by flow cytometry. Box blots show **(A)** MHC class I and **(B)** MHC class II expression on BDCA-2^+^ pDCs. Box central horizontal lines indicate medians, box borders represent IQR, whiskers indicate minima and maxima. Significance levels were ***p < 0.0005 and *p < 0.05. BDCA-2, blood dendritic cell antigen 2; IQR, interquartile range; MHC, major histocompatibility complex; n.s., not significant; pDC, plasmacytoid dendritic cell.

One of the natural drivers and adjuvants for the development of antigen-specific T cell responses are dendritic cells. Among these naturally occurring plasmacytoid dendritic cells (pDCs) play a particularly important role for the defense of viral infections due to their capacity to cross-present antigens and provide strong co-stimulatory signals to both T helper and cytotoxic T cells (39, 40). Accordingly, we found antigen-presenting MHC molecules to be upregulated with a particularly strong effect on MHC class II (**Figures 5A, B**). In contrast, the effect of booster vaccination on co-stimulatory molecule expression was more heterogeneous than in B cells with CD86 rather remaining unaltered or even being suppressed, while CD154 (CD40 ligand) being strongly and significantly enhanced (**Supplementary Figures 4A, B**). Since pDC can also exhibit immunoregulatory potential in certain situations, we analyzed them for intracellular expression of known mediators of immunosuppression as well. These data revealed a significant upregulation of GrB, but not IL-10 in pDC from those cohorts consisting of two or three mRNA booster vaccinations (**Supplementary Figures 5A, B**).

Surprisingly and in contrast to our findings in B cells, we observed a decrease rather than an increase in the frequency of peripheral pDCs within the first three months after booster vaccination with mRNA vaccines (0.36% - 0.44% before 1^st^ vaccination, 0.26% - 0.32% after booster vaccination) (**Supplementary Figure 6A**). On first sight this may appear contradictory to our finding that pDCs are activated after booster vaccination. On the other hand however, this observation may indicate pDC migration from the peripheral blood into lymph nodes, where they can process and express antigens including SARS-CoV-2-specific oligopeptides on their surface, thereby presenting them to antigen-specific T cells. This view is supported by stable or even enhanced peripheral pDC viability after booster vaccination (84.8% - 92.9% before 1^st^ vaccination, 92.5% - 94.0% after booster vaccination) (**Supplementary Figure 6B**).

**Figure 6:**
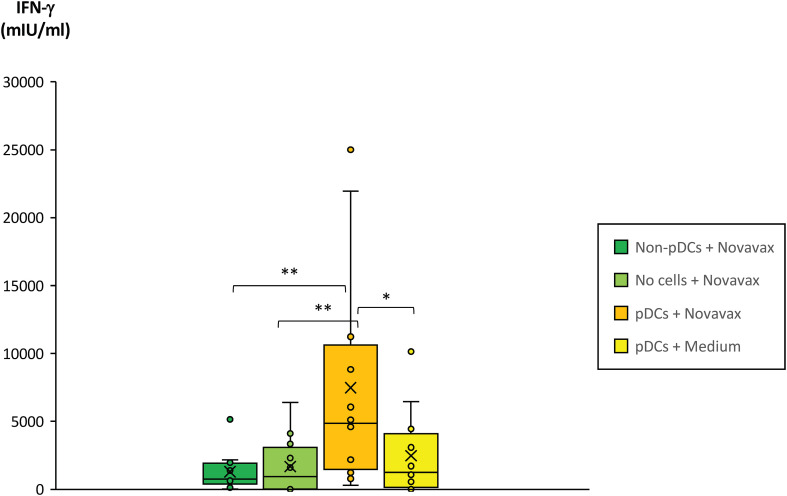
Impact of pDCs pulsed with SARS-CoV-2 peptide antigens on the SARS-CoV-2-specific T cell response of fully vaccinated individuals. Plasmacytoid dendritic cells (pDCs) from 12 healthy and fully vaccinated individuals were isolated and pre-incubated on a 96-well plate for 3 days in the presence of 10ng/ml IL-3 and in the presence or absence of 0.8µg/ml SARS-CoV-2 peptide antigen (Novavax). Non-pDCs incubated in the presence of IL-3 and Novavax served as control. On day 2, 2.5µg/ml ODN 2006 was added to all wells to induce maturation of pDCs. On day 3, PBMCs from the corresponding donors were freshly isolated and co-incubated with the pre-incubated samples at a cellular ratio of 40:1. PBMCs incubated in cell-free medium containing 0.8µg/ml Novavax served as an additional control (bright green bar). After 20–24 hours co-incubation, PBMC supernatants were harvested and IFN-γ concentrations quantified by a specific ELISA. Box blots show IFN-γ concentrations in the PBMC supernatants. Box central horizontal lines indicate medians, box borders represent IQR, whiskers indicate minima and maxima. Significance levels were **p < 0.005 and *p < 0.05. IFN-γ, interferon-gamma; IQR, interquartile range; PBMC, peripheral blood mononuclear cells.

To test the above-mentioned hypothesis, pDCs from healthy triple-vaccinated donors were isolated, loaded with SARS-CoV-2-specific peptides from the approved COVID-19 peptide vaccine Novavax and matured by incubation with the TLR agonist ODN 2006 as described in Materials and Methods. Then, matured pDCs were co-incubated with freshly isolated PBMCs from the corresponding donors (autologous incubation) for 24 hours. Finally, culture supernatants were harvested and SARS-CoV-2-specific IFN-γ secretion by T cells was measured by ELISA. These experiments showed that the presence of pDCs loaded with SARS-CoV-2-specific peptides strongly and significantly enhanced SARS-CoV-2- specific T cell responses in vaccinated donors (**Figure 6**). In contrast, or SARS-CoV-2-specific peptides alone or in combination with non-pDCs did not have comparable effects.

### Protection against breakthrough infections after booster vaccination with an mRNA vaccine does not depend on the number of mRNA shots before booster vaccination

**Figure 7:**
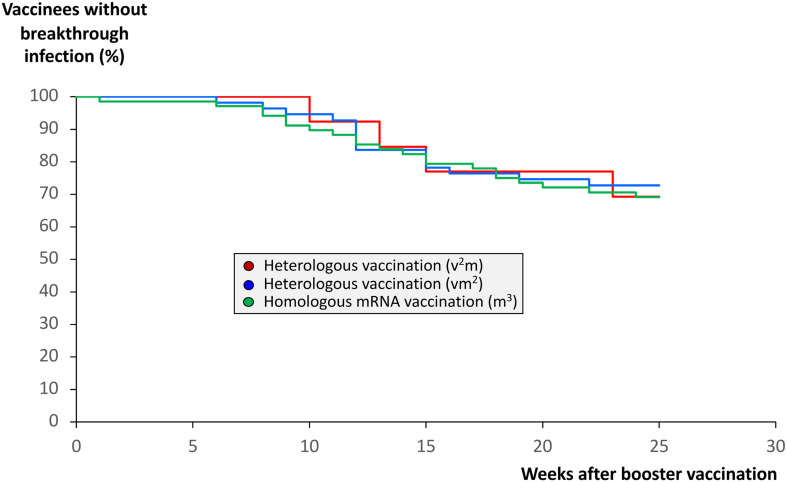
Impact of anti-SARS-CoV-2 vaccination regimes on the frequency of COVID-19 breakthrough infections. Infection status from 68 individuals having received a regime with three mRNA vaccines (m^3^, green line), from 55 individuals having received a regime with one vector and two mRNA vaccines (vm^2^, blue line), and from 13 individuals having received a regime with two vector and one mRNA vaccine (v^2^m, red line), was followed up until 25 weeks after mRNA booster vaccination (3^rd^ vaccination). In case COVID-19 breakthrough infections occurred, they were confirmed by nucleic acid testing (NAT). The Kaplan-Meier curves indicate no significant differences between the homologous and the two heterologous anti-SARS-CoV-2 vaccination regimes regarding the frequency of COVID-19 breakthrough infections.

As outlined above, we found significantly higher anti-SARS-CoV-2 IgG titers, Omicron neutralization capacities as well as pan-T cell IFN-γ secretion responses in those vaccination regimes consisting of at least two mRNA shots. We therefore hypothesized that vaccination regimes consisting of two or more mRNA shots may also provide a stronger or more durable protection against breakthrough infections compared with the vaccination regime with one mRNA booster only. We thus analyzed the three cohorts for the occurrence of breakthrough infections after booster vaccination. Surprisingly, we found that at least up to 25 weeks after booster vaccination the percentage of such infections was almost identical among the three vaccination groups (**Figure 7**). Comparison of SARS-CoV-2 antibody and neutralization titers as well as SARS-CoV-2-specific IFN-γ release by T cells before and after breakthrough infection showed a significant increase in the mentioned parameters after infection, which was particularly strong in those cohorts with two and three mRNA booster vaccinations (**Supplementary Figures 7A–C**). No significant differences were detected between the three cohorts.

## Discussion

In the present follow-up study to our initial study published in 2021 (10), we directly compared three vaccination regimes with regard to various aspects of SARS-CoV-2-specific humoral and cellular immunity, including the strength of the response, its development over a longer period of time, and its impact on the occurrence of COVID-19 breakthrough infections. The data we collected covered a time period of up to 68 weeks after booster vaccination and included one vaccination cohort receiving a homologous mRNA vaccination regime (m3, either Comirnaty or Spikevax), one cohort receiving a heterologous vaccination regime involving one vaccination with the vector vaccine Vaxzevria as well as two vaccinations with an mRNA vaccine (vm2), and one cohort receiving a heterologous vaccination regime involving two vaccinations with the vector vaccine Vaxzevria as well as one booster with an mRNA vaccine (v2m). As described by a number of independent studies, the cornerstone of SARS-CoV-2 immunity elicited by mRNA booster vaccination is a robust humoral immune response (21, 22, 24, 25, 44, 45). Data from our current study indicated that IgG titers peaked between 4 and 10 weeks after the booster dose, reaching averages of 4079 BAU/ml in the homologous mRNA (m3) cohort, compared with 2454 BAU/ml and 1502 BAU/ml in the heterologous vm2 and v2m cohorts, respectively. These levels remained stable for up to 64 weeks following first vaccination, highlighting the durability of the immune response. The significantly elevated IgG levels across all regimes confirm and underscore the effectiveness of mRNA boosters in stimulating antibody production and neutralization capacity (10, 29–31, 44, 45), independently of the type of vaccine, which initiated the immunization regime.

While the neutralization capacity against the wildtype virus was nearly 100% across cohorts, the capacity to neutralize VOC like Omicron varied significantly. Again, the homologous mRNA vaccination regime (m3) appeared to provide superior Omicron neutralization with 57% protection compared with the heterologous vaccination regimes vm2 with 22% and v2m with 6% protection. This finding suggests consistent mRNA exposure may generate antibodies capable of addressing potential antigenic drift in the course of viral evolution (27, 28). Importantly, the differences identified may not only reflect quantitative but also qualitative aspects of the immune response, as mRNA boosters appeared to refine the affinity and breadth of antibodies through B cell maturation. The implications of these findings may extend to emerging variants, where high-affinity antibodies may offer partial cross-protection (10, 29–31). This hypothesis was particularly supported by our surprising observation that some non-directional cross-protection against the Omicron variant already occurred in one heterologous vaccination cohort (vm2) even months before this VOC had broadly evolved and spread.

In this context, the role of B cells, which represent the adaptive immune system’s enduring capacity to respond rapidly to re-exposure (21, 22), deserves particular attention. Following booster vaccination, these cells exhibited significant activation, indicated by upregulation of MHC class I/II and CD86 expression. These markers are associated with antigen presentation and co-stimulation, enabling effective interactions with helper T cells to sustain antibody production. Additionally, B cells undergo affinity maturation in germinal centers (27, 28), enhancing their ability to produce high-affinity antibodies against SARS-CoV-2 and its variants (10, 29–31). The dynamics of such B cell remodeling were particularly evident in response to the Omicron variant. The observed shifts in clonal composition and the spontaneous emergence of high-affinity antibodies against not-yet-existing variants (as shown for Omicron in our study) demonstrate that mRNA re-vaccinations are not only able to restore waning immunity but also to refine the immune repertoire, particularly in a heterologous vaccination setting (10, 14–19). This aligns with longitudinal studies showing that successive antigenic exposures improve B cell functionality (10, 29–31, 44, 45). A homologous mRNA vaccination on the other hand, which provides repeated exposure to the same antigenic format, appears to further optimize this process, supporting its general use in booster strategies (10, 17–19).

Another cell type primarily involved in antigen recognition and presentation during viral infections is the so-called plasmacytoid dendritic cell (pDC). In contrast to other dendritic cell types, precursors of pDCs are permanently circulating and patrolling the peripheral blood system (36, 37). By linking the innate and adaptive arms of immunity, they are considered central to antiviral immune response including the response to vaccinations (39, 40). They are unique in their capacity to produce type I interferons, which activate antiviral pathways and enhance immune cell functionality (36). In our study we found that the antigen-presenting capacity of pDCs was significantly enhanced after booster vaccination with mRNA vaccines as well. We also observed a transient reduction in peripheral pDC frequencies after booster vaccination. This was likely due to migration to secondary lymphoid tissues, a pathophysiological response after encounter of pDCs with certain pathogen-associated molecular patterns, which are also contained in mRNA vaccines and which can activate TLR8 in these cells (5, 46, 47). With functional *in-vitro* assays we demonstrated that pDCs loaded with SARS-CoV-2-specific peptides strongly and significantly enhanced SARS-CoV-2- specific T cell responses in vaccinated donors. Moreover, donors showed an upregulation of MHC class II and CD154 expression on pDCs post-booster vaccination, potentially enabling antigen-presentation combined with robust activation of antigen-specific T cells. Accordingly, we demonstrated that activation of CD4^+^ helper and CD8^+^ cytotoxic T cells was significantly enhanced by pDCs post-booster, with mRNA-based regimes eliciting the strongest responses. IFN-γ production, a hallmark of T cell activity, was markedly elevated in these cohorts, further supporting the importance of T cell-mediated immunity in countering SARS-CoV-2 (29, 30, 33, 48–50).

Besides their role in antigen presentation and T cell activation, pDCs may indirectly contribute to B cell activation as well. By creating a cytokine-rich environment mainly consisting of type I interferons, they support germinal center formation and differentiation of B cells into plasma cells (36, 51). This dual functionality also positions pDCs as central components of the immune response to SARS-CoV-2 vaccination. While pDC-activated CD8^+^ T cells directly target and eliminate infected cells, CD4^+^ T cells facilitate B cell activation and antibody production by providing necessary co-stimulatory signals (36). The durability of these responses, sustained and documented for up to 60 weeks post-vaccination in our study, underscores their resilience against waning antibody levels and emerging variants. Since T cells often recognize conserved viral epitopes, their response is less vulnerable to antigenic drift, thereby ensuring continued efficacy even as the virus evolves (48, 49). This aspect of T cell-mediated immunity is particularly relevant in the context of Omicron and its subvariants, which have demonstrated significant immune escape (4, 50).

Emerging evidence highlights a dual role for pDCs in modulating immune responses through both immunogenic and regulatory mechanisms. Tolerogenic properties of pDCs can be shaped by cytokines such as IL-3 and IL-10, leading to the production of granzyme B (GrB), which has been shown to suppress T cell proliferation in a perforin-independent, contact-dependent manner, thereby mimicking features of regulatory T cells (43)​. Furthermore, tolerogenic dendritic cells including pDCs, may promote peripheral tolerance via inhibitory molecules like PD-L1 and secreted factors such as IL-10, TGF-β, and IDO, as well as through epigenetic regulation of immunomodulatory genes (52). These mechanisms position pDCs as key contributors to immunological balance, particularly under steady-state or vaccine conditions. A comprehensive analysis throughout the three cohorts of our study revealed a significant upregulation of GrB, but not IL-10 in pDCs from the cohorts consisting of two or three mRNA booster vaccinations. This may point to a potential immunoregulatory role of pDCs during mRNA vaccinations, with particular significance for potential autoimmune adverse reactions (53). Overall, our findings regarding pDCs further support their pivotal role for the efficacy of mRNA vaccines. Future vaccination strategies may therefore even consider the application of mRNA vaccines along with cell preparations of viable pDCs. On the one hand such combinations could allow the development of stronger immune responses particularly in cases where mRNA vaccines alone are not efficient enough to trigger strong protection against certain infectious diseases (54). On the other hand, similar vaccination approaches may be tested in other diseases such as certain malignancies (55, 56) or in individuals with impaired immunogenicity (57, 58).

An intriguing aspect of our study was the observation that breakthrough infection rates were comparable across cohorts up to 25 weeks after booster vaccination, despite significant differences in antibody titers. This finding underscores the multifaceted nature of vaccine-induced immunity, where humoral and cellular responses collectively contribute to protection (29, 30, 33). We therefore hypothesize that cellular immunity, particularly T cell activity and the supportive action of dendritic cell types like pDC, likely compensated for the lower antibody levels in heterologous vaccination regimes, thereby preventing significantly worse outcomes than in the homologous regime. Thus, the synergy between T cells, B cells, and pDCs highlights the importance of eliciting an integrated immune response for mitigating certain viral infections such as with SARS-CoV-2. Even in the presence of waning antibody titers, robust T cell responses and effective antigen presentation by pDCs ensure a degree of protection against severe disease, particularly in those vaccination regimes containing two or three mRNA booster vaccinations (29, 30, 33). Importantly, recent meta-analyses and longitudinal studies also indicated comparable vaccination efficacy across different three-dose SARS-CoV-2 vaccination strategies, whether homologous or heterologous (59–63). These studies also support our hypothesis, that the compensatory mechanisms described above may be crucial for maintaining population immunity as booster intervals are extended or as new variants emerge.

The results of our study support a growing body of evidence pointing into a similar direction with regard to future vaccination strategies. Enhancing the activation and functionality of pDCs and T cells could provide broader and more durable protection, not only against viral but also other infectious agents (64). Variant-adapted boosters, incorporating antigens that target conserved viral epitopes, may offer improved efficacy against immune-evasive strains (46, 64). Moreover, the development of overarching vaccines such as a pan-coronavirus vaccine, capable of addressing multiple SARS-CoV-2 lineages and related coronaviruses, represents another interesting avenue for research (65). Personalized vaccination strategies, tailored to individual immune profiles and risk factors, potentially incorporating cell therapeutic strategies, may also enhance overall effectiveness of vaccines (66).

While our study provides important additional insights into long-term immunity of vaccinated individuals, several limitations may be addressed in future work. Functional *in vitro* assays stimulating B cells with spike protein could reveal differentiation into memory or plasma cells and clarify responses across vaccine regimens. T cell phenotyping and cytokine profiling beyond IFN-γ would refine our understanding of cellular immunity. Given prior reports of pDC involvement in plasma cell development and T cell-independent B cell responses, co-culture or pDC-depletion experiments could clarify their functional role. Assessing migratory markers such as CCR7, CD62L, and integrins β1/β2 could support our hypothesis of pDC redistribution to lymphoid tissues. Direct measurement of spike antigen uptake by pDCs would strengthen evidence for their antigen-presenting capacity. Finally, recent organoid data showing pDC-mediated IFN-α responses to vector vaccines suggest similar pathways may shape mRNA-induced immunity.

In conclusion, we demonstrated that mRNA booster vaccinations elicit robust and sustained immune responses by activating both humoral and cellular pathways. Homologous mRNA regimes (m3) consistently achieved superior antibody titers and neutralization capacities, particularly against VOCs like Omicron. The activation of B cells and pDCs, as well as the adjuvant effect of pDCs on antigen-specific T cell activation underscore the importance of these cells in maintaining immunity over time. Notably, the robust IFN-γ-driven T cell responses observed in cohorts with two or more mRNA shots highlight the resilience of cellular immunity in addressing immune-evasive variants. Despite differences in antibody levels, comparable breakthrough infection rates across cohorts emphasize the role of cellular immunity in mitigating severe disease. These findings underscore the need for a multifaceted approach to vaccine design, integrating robust stimulation of B cells, pDCs and T cells. Variant-specific and pan-coronavirus booster formulations, potentially combined with cell-based adjuvants, hold promise for enhancing long-term protection despite ongoing viral mutations in future pandemic situations.

## Data Availability

The raw data supporting the conclusions of this article will be made available by the authors, without undue reservation.
