# Supplementary material for: Effective cellular and neutralizing immunity against SARS-CoV-2 after mRNA booster vaccination is associated with pDC and B cell activation
Source: Front Immunol. 2025 May 12;16:1580448. doi: 10.3389/fimmu.2025.1580448 (PMC12104250; doi:10.3389/fimmu.2025.1580448)

	Quantitative serological testing			Flow cytometric analysis		
**Vaccination regime**	**Heterologous (v^2m)**	**Heterologous (vm^2)**	**Homologous (m^3)**	**Heterologous (v^2m)**	**Heterologous (vm^2)**	**Homologous (m^3)**
**Vaccine types**	**2x vector, 1x mRNA**	**1x vector, 2x mRNA**	**3x mRNA**	**2x vector, 1x mRNA**	**1x vector, 2x mRNA**	**3x mRNA**
**Number of individuals**	13	55	68	10	22	27
**Number females (%)**	7 (53.8%)	43 (78.2%)	53 (77.9%)	7 (70%)	19 (86.4%)	22 (81.5%)
**Number males (%)**	6 (46.2%)	12 (21.8%)	15 (22.1%)	3 (30%)	3 (13.6%)	5 (18.5%)
**Average age (range)**	57 (23-78)	43 (20-64)	48 (18-65)	56 (23-78)	45 (22-64)	48 (22-64)

Supplementary Table 1. Age and gender characteristics of vaccination cohorts. In the present study, three different vaccination schedules were compared among each other in terms of serological and cellular immune responses. Vaccination schedules included either a homologous vaccination regime consisting of three vaccinations with an mRNA vaccine (m^3), a heterologous vaccination regime consisting of one vaccination with a vector vaccine followed by two mRNA vaccinations (vm^2), or a heterologous vaccination regime consisting of two vaccinations with a vector vaccine, followed by one mRNA vaccination (v^2m). Methods used for quantitative serological testing included an anti-SARS-CoV-2-IgG ELISA and enzymatic neutralization tests for the wildtype and the Omicron variant of the SARS-CoV-2 RBD. Methods used for the characterization of the cellular immune response were based on flow cytometric analyses and ELISA-based detection of IFN-γ secretion by SARS-CoV-2-specific T cells. Abbreviation: RBD = receptor binding domain.

Supplementary Figure 1

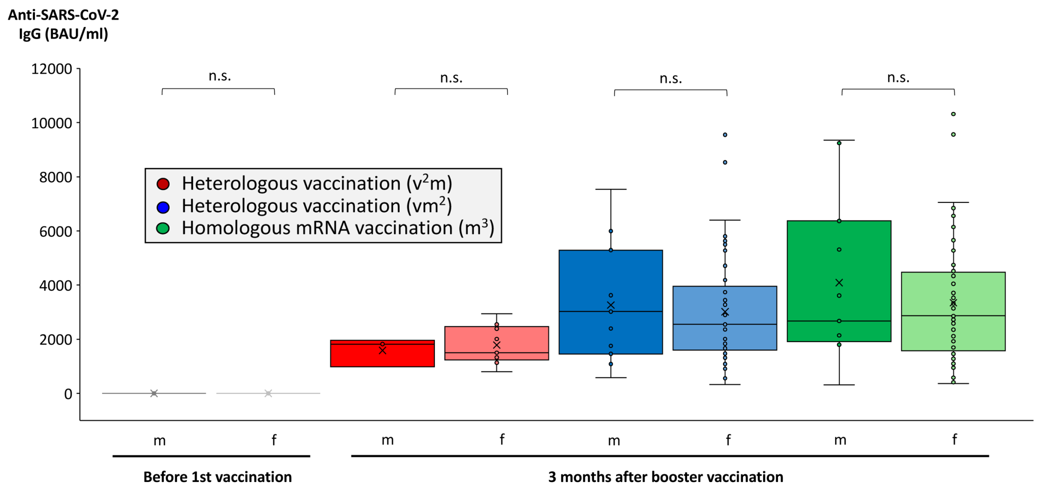

Supplementary Figure 1. Gender-specific comparison of anti-SARS-CoV-2 IgG titers in homologous and heterologous vaccination regimes

Serum samples from up to 64 individuals having received a regime with three mRNA vaccines (m^3, green bars), up to 53 individuals having received a regime with one vector and two mRNA vaccines (vm^2, blue bars), and up to 13 individuals having received a regime with two vector and one mRNA vaccine (v^2m, red bars), were collected before 1st vaccination and 3 months after mRNA booster vaccination (3rd vaccination) as indicated. Box blots show anti-SARS-CoV-2 IgG titers , differentiated for both male (m) and female (f) donors. Box central horizontal lines indicate medians, box borders represent IQR, whiskers indicate minima and maxima. Abbreviations: IQR = interquartile range, n.s. = not significant.

Supplementary Figure 2A

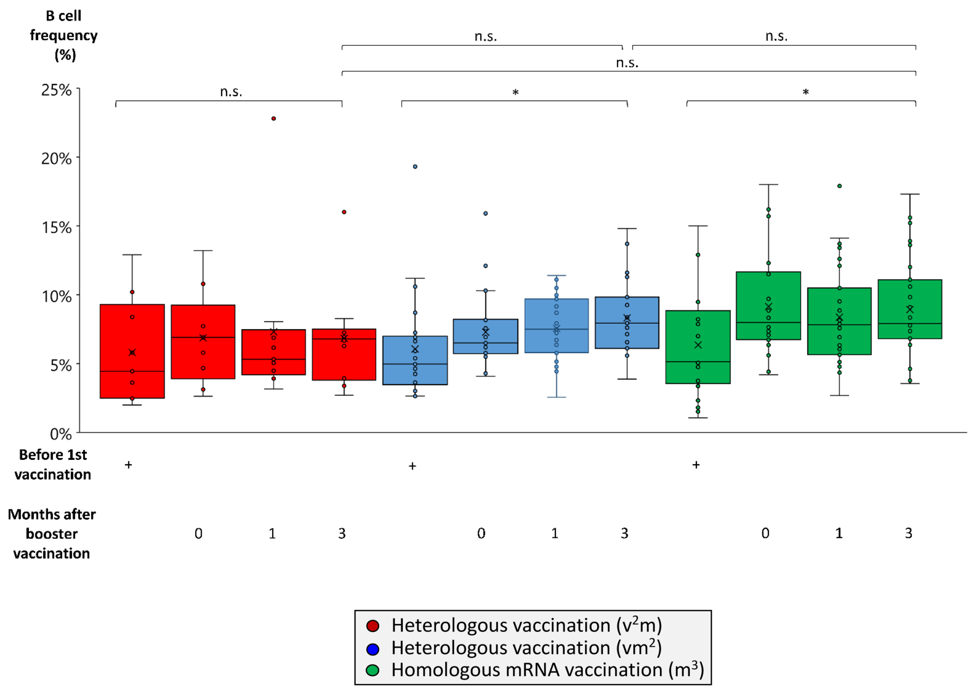

Supplementary Figure 2B

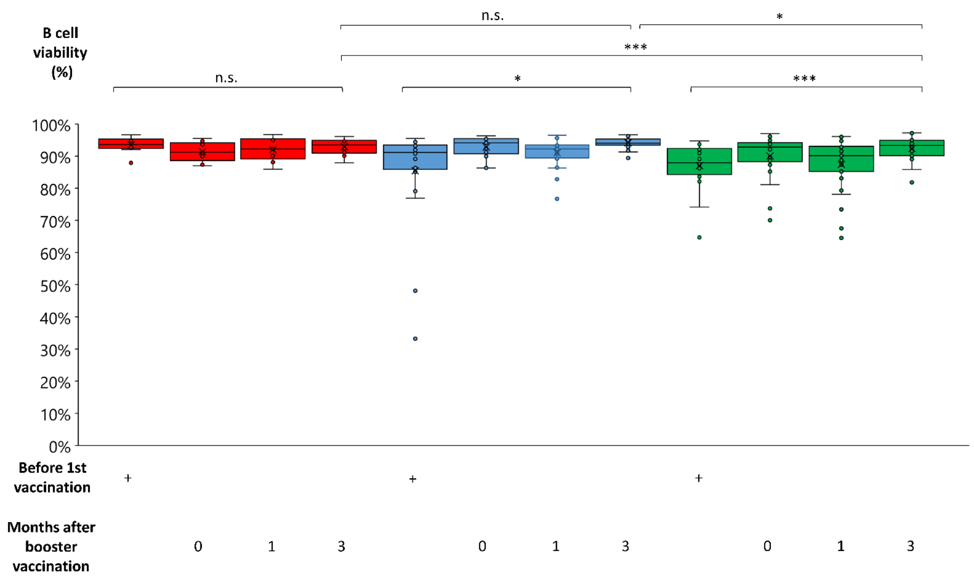

Supplementary Figure 2. Comparison of B cell frequency and viability in individuals undergoing various anti-SARS-CoV-2 vaccination regimes

Heparin blood samples from up to 27 individuals having received a homologous vaccination regime with three mRNA vaccines (m^3, green bars), up to 22 individuals having received a heterologous vaccination regime with one vector and two mRNA vaccines (vm^2, blue bars), and up to 10 individuals having received a heterologous vaccination regime with two vector and one mRNA vaccine (v^2m, red bars), were collected before 1st vaccination and 0, 1 and 3 months after mRNA booster vaccination (3rd vaccination) as indicated. PBMCs were isolated and cryopreserved until further use. Then, PBMCs were thawed, B cells stained as described in the Materials & Methods section and analyzed by flow cytometry. Box blots show (**A**) the frequency and (**B**) the viability of $CD19^+$ B cells within the PBMC fraction. Box central horizontal lines indicate medians, box borders represent IQR, whiskers indicate minima and maxima. Significance levels were *** $p < 0.0005$ and * $p < 0.05$. Abbreviations: IQR = interquartile range, MHC = major histocompatibility complex, n.s. = not significant.

Supplementary Figure 3A

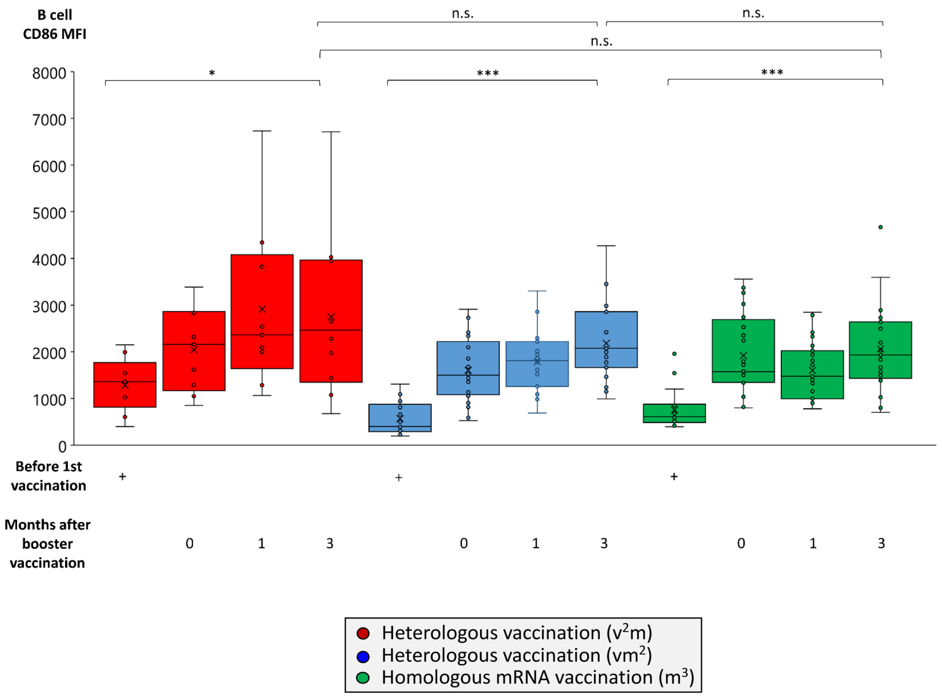

Supplementary Figure 3B

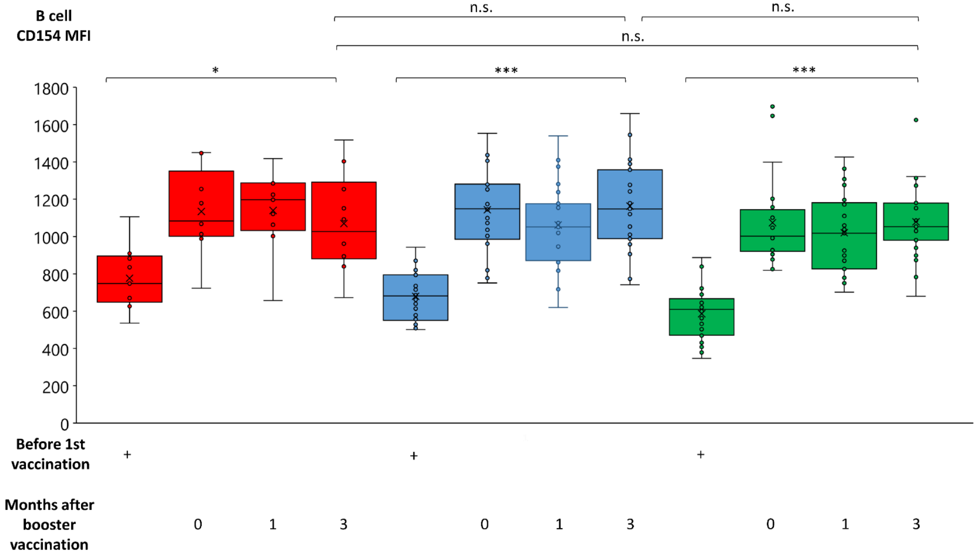

Supplementary Figure 3. Comparison of costimulatory molecule expression in B cells from individuals undergoing various anti-SARS-CoV-2 vaccination regimes
Heparin blood samples from up to 27 individuals having received a regime with three mRNA vaccines (m^3, green bars), up to 22 individuals having received a regime with one vector and two mRNA vaccines (vm^2, blue bars), and up to 10 individuals having received a regime with two vector and one mRNA vaccine (v^2m, red bars), were collected before 1st vaccination and 0, 1 and 3 months after mRNA booster vaccination (3rd vaccination) as indicated. PBMCs were isolated and cryopreserved until further use. Then, PBMCs were thawed, B cells stained as described in the Materials & Methods section and analyzed by flow cytometry. Box blots show (**A**) CD86 and (**B**) CD154 (CD40 ligand) expression in $CD19^+$ B cells. Box central horizontal lines indicate medians, box borders represent IQR, whiskers indicate minima and maxima. Significance levels were *** $p < 0.0005$, ** $p < 0.005$ and * $p < 0.05$. Abbreviations: IQR = interquartile range, MHC = major histocompatibility complex, n.s. = not significant.

Supplementary Figure 4A

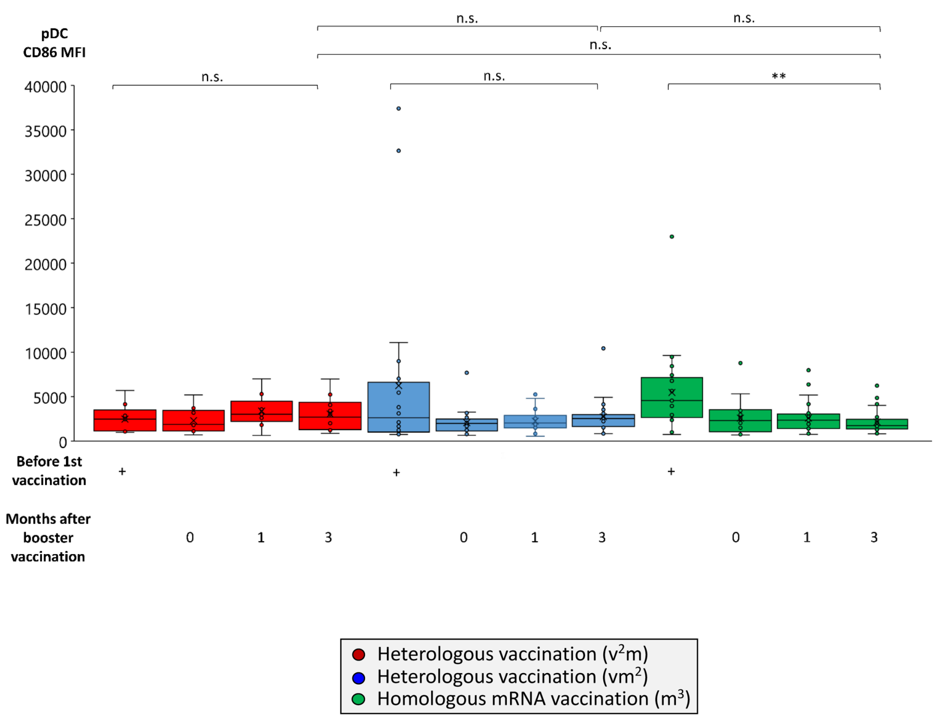

Supplementary Figure 4B

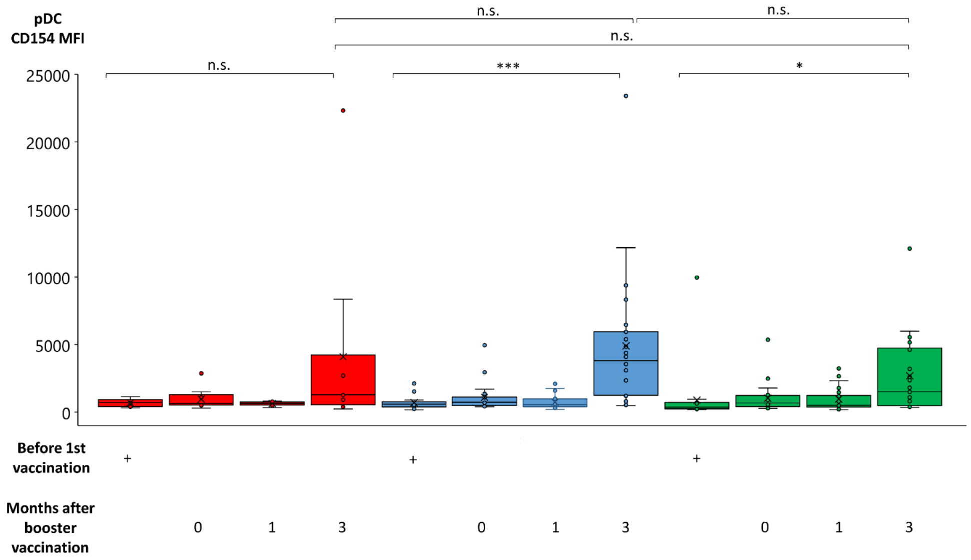

Supplementary Figure 4. Comparison of costimulatory molecule expression in pDCs from individuals undergoing various anti-SARS-CoV-2 vaccination regimes

Heparin blood samples from up to 27 individuals having received a regime with three mRNA vaccines (m^3, green bars), up to 22 individuals having received a regime with one vector and two mRNA vaccines (vm^2, blue bars), and up to 10 individuals having received a regime with two vector and one mRNA vaccine (v^2m, red bars), were collected before 1st vaccination and 0, 1 and 3 months after mRNA booster vaccination (3rd vaccination) as indicated. PBMCs were isolated and cryopreserved until further use. Then, PBMCs were thawed, pDCs stained as described in the Materials & Methods section and analyzed by flow cytometry. Box blots show (**A**) CD86 and (**B**) CD154 (CD40 ligand) expression on BDCA-2$^+$ pDCs. Box central horizontal lines indicate medians, box borders represent IQR, whiskers indicate minima and maxima. Significance levels were *** $p < 0.0005$, ** $p < 0.005$ and * $p < 0.05$. Abbreviations: BDCA-2 = blood dendritic cell antigen 2, IQR = interquartile range, MHC = major histocompatibility complex, n.s. = not significant, pDC = plasmacytoid dendritic cell.

Supplementary Figure 5A

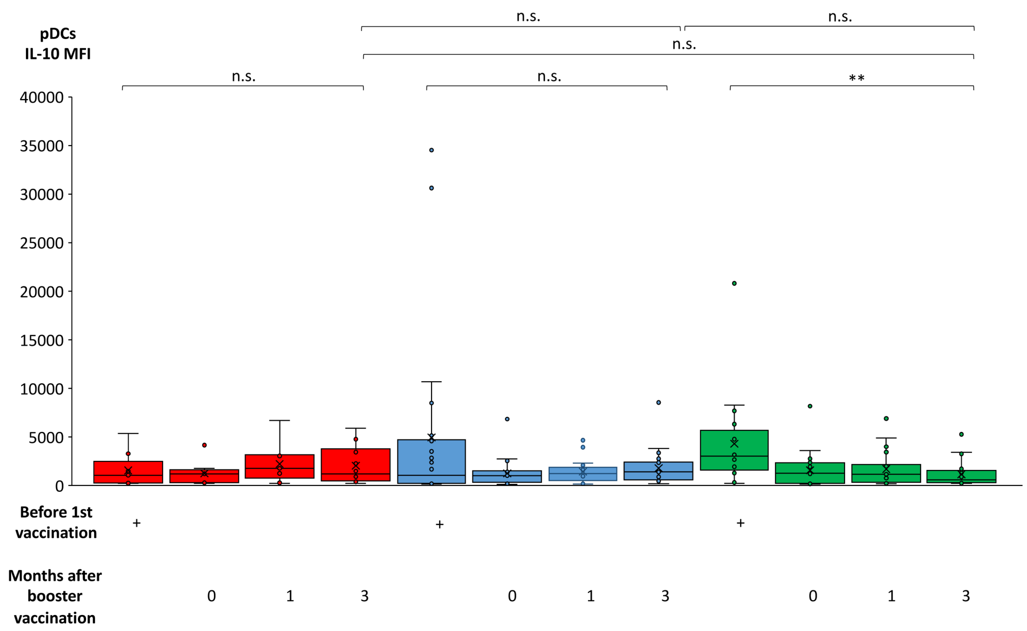

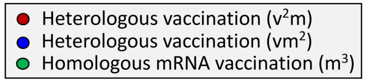

Supplementary Figure 5B

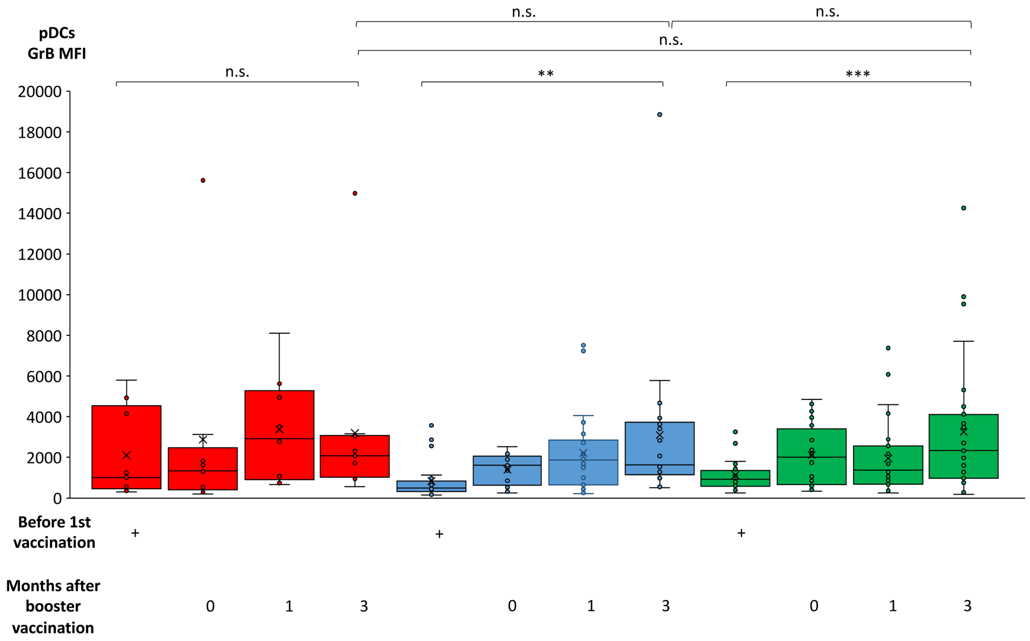

Supplementary Figure 5 Comparison of immunoregulatory molecule expression in pDCs from individuals undergoing various anti-SARS-CoV-2 vaccination regimes

Heparin blood samples from up to 27 individuals having received a regime with three mRNA vaccines (m^3, green bars), up to 22 individuals having received a regime with one vector and two mRNA vaccines (vm^2, blue bars), and up to 10 individuals having received a regime with two vector and one mRNA vaccine (v^2m, red bars), were collected before 1st vaccination and 0, 1 and 3 months after mRNA booster vaccination (3rd vaccination) as indicated. PBMCs were isolated and cryopreserved until further use. Then, PBMCs were thawed, fixed and permeabilized, and pDC stained and analyzed by flow cytometry as described in the Materials & Methods section. Box blots show (**A**) IL-10 and (**B**) GrB expression in BDCA-2^+ pDCs. Box central horizontal lines indicate medians, box borders represent IQR, whiskers indicate minima and maxima. Significance levels were *** $p < 0.0005$ and ** $p < 0.005$. Abbreviations: BDCA-2 = blood dendritic cell antigen 2, GrB = granzyme B, IL-10 = Interleukin 10, IQR = interquartile range, MHC = major histocompatibility complex, n.s. = not significant, pDC = plasmacytoid dendritic cell.

Supplementary Figure 6A

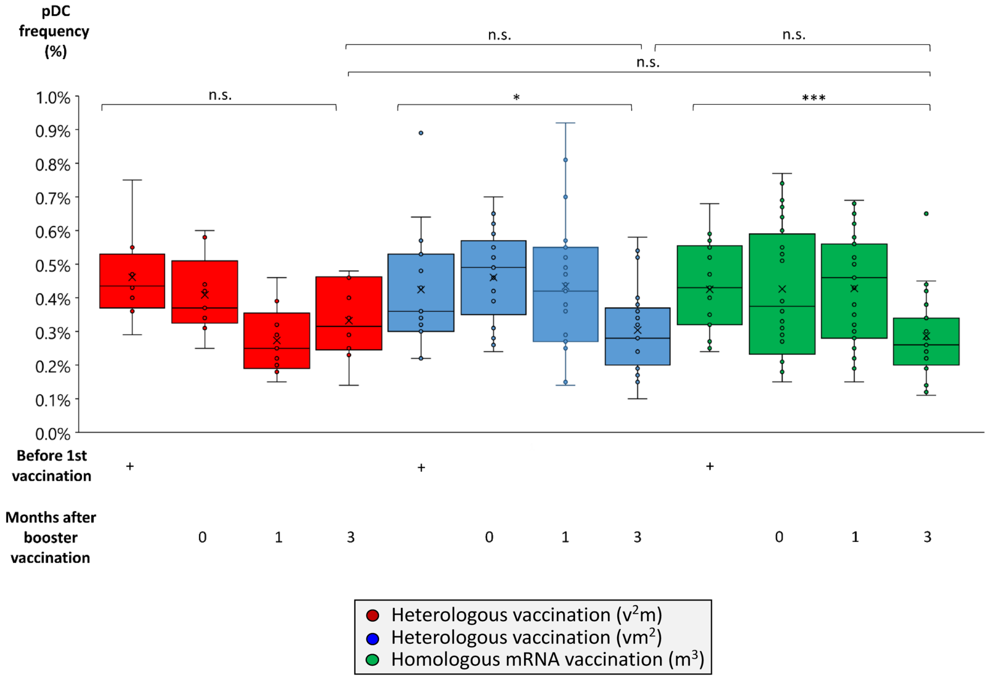

Supplementary Figure 6B

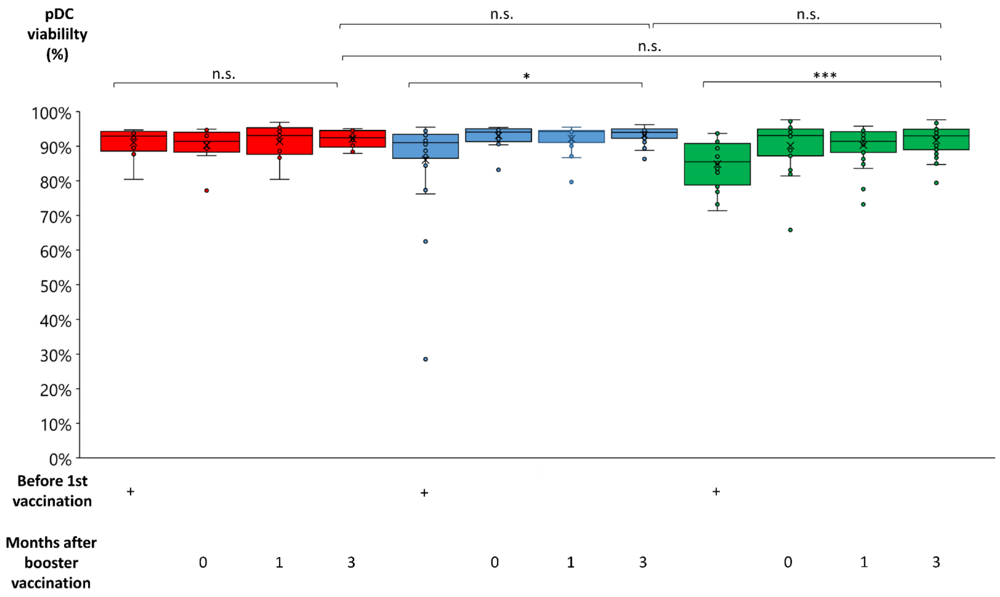

Supplementary Figure 6. Comparison of pDC frequency and viability in individuals undergoing various anti-SARS-CoV-2 vaccination regimes

Heparin blood samples from up to 27 individuals having received a regime with three mRNA vaccines (m^3, green bars), up to 22 individuals having received a regime with one vector and two mRNA vaccines (vm^2, blue bars), and up to 10 individuals having received a regime with two vector and one mRNA vaccine (v^2m, red bars), were collected before 1st vaccination and 0, 1 and 3 months after mRNA booster vaccination (3rd vaccination) as indicated. PBMCs were isolated and cryopreserved until further use. Then, PBMCs were thawed, pDCs stained as described in the Materials & Methods section and analyzed by flow cytometry. Box blots show (**A**) the frequency and (**B**) the viability of BDCA-2$^+$ pDCs within the PBMC fraction. Box central horizontal lines indicate medians, box borders represent IQR, whiskers indicate minima and maxima. Significance levels were *** $p < 0.0005$ and * $p < 0.05$. Abbreviations: BDCA-2 = blood dendritic cell antigen 2, IQR = interquartile range, MHC = major histocompatibility complex, n.s. = not significant, pDC = plasmacytoid dendritic cell.

Supplementary Figure 7A

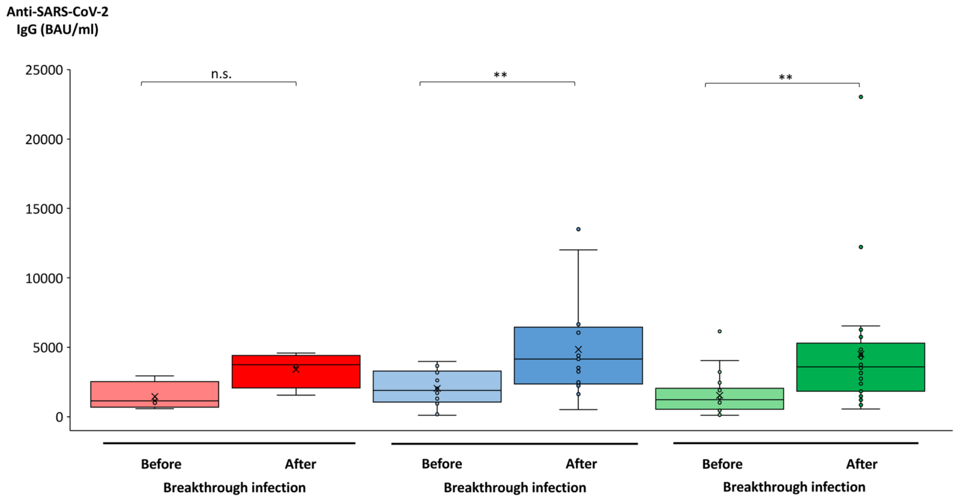

Supplementary Figure 7. Comparison of antibody and neutralization titers as well as SARS-CoV-2-specific T cell responses in homologous and heterologous anti-SARS-CoV-2 vaccination regimes before and after breakthrough infection

Within the observation period 25 individuals having received a regime with three mRNA vaccines (m^3, green bars), 17 individuals having received a regime with one vector and two mRNA vaccines (vm^2, blue bars), and 4 individuals having received a regime with two vector and one mRNA vaccine (v^2m, red bars), suffered a breakthrough infection with SARS-CoV-2. SARS-CoV-2 antibody and neutralization titers as well as SARS-CoV-2-specific IFN-γ release by T cells from the last blood sampling before breakthrough infection as well as the first blood sampling after breakthrough infection were summarized and compared. Box blots show (**A**) anti-SARS-CoV-2 IgG titers, (**B**) Omicron neutralization capacities, and (**C**) median IFN-γ release in the various vaccination regimes. Box central horizontal lines indicate medians, box borders represent IQR, whiskers indicate minima and maxima. Significance levels were *** $p < 0.0005$, ** $p < 0.005$ and * $p < 0.05$. Abbreviations: IQR = interquartile range, n.s. = not significant.

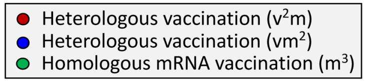

Supplementary Figure 7B

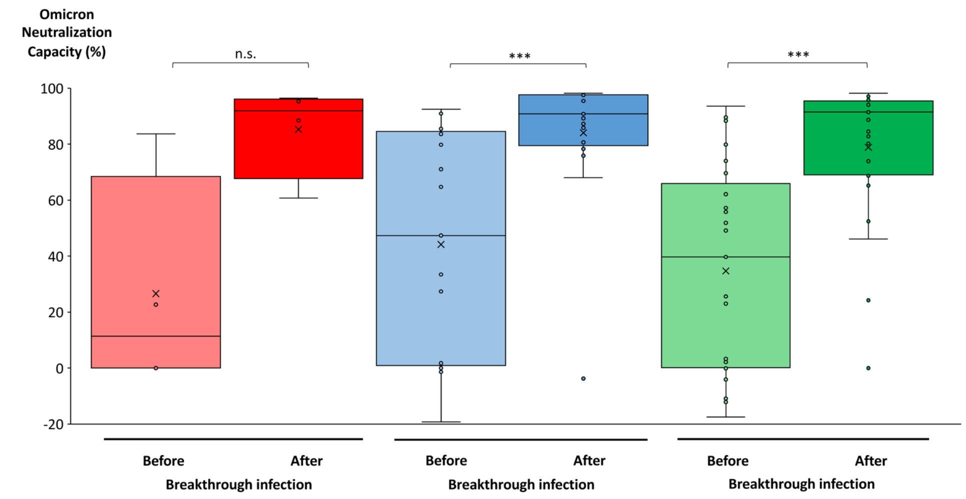

Supplementary Figure 7C

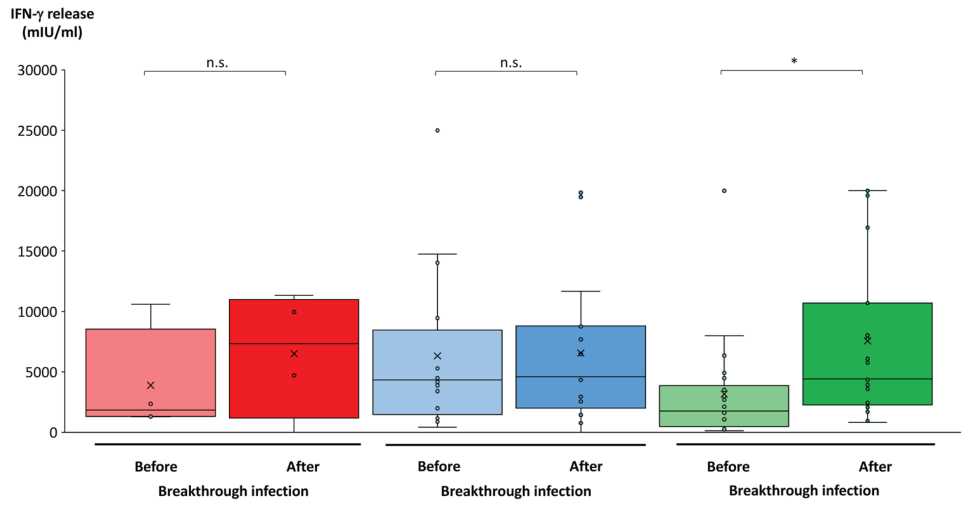

Supplement: Supplementary file 1 [file DataSheet1.pdf]
